# CPCGI Alleviates Neural Damage by Modulating Microglial Pyroptosis After Traumatic Brain Injury

**DOI:** 10.1111/cns.70322

**Published:** 2025-03-09

**Authors:** Lu‐Lu Yu, Lei Sun, Ting‐Ting Yu, An‐Chen Guo, Jian‐Ping Wu, Jun‐Min Chen, Qun Wang

**Affiliations:** ^1^ Department of Neurology Beijing Tiantan Hospital, Capital Medical University Beijing China; ^2^ China National Clinical Research Center for Neurological Diseases Beijing China; ^3^ Department of Neurology Zhengzhou University People's Hospital Zhengzhou China; ^4^ Department of Neurology The First Affiliated Hospital of Soochow University Suzhou China; ^5^ Beijing Institute of Brain Disorders, Collaborative Innovation Center for Brain Disorders Capital Medical University Beijing China; ^6^ Beijing Key Laboratory of Drug and Device Research and Development for Cerebrovascular Diseases Beijing China; ^7^ Advanced Innovation Center for Human Brain Protection Capital Medical University Beijing China; ^8^ Department of Neurology The First Affiliated Hospital of Zhengzhou University Zhengzhou China

**Keywords:** compound porcine cerebroside ganglioside injection, gasdermin D, neuroinflammation, NLRP3 inflammasomes, pyroptosis, traumatic brain injury

## Abstract

**Background:**

Traumatic brain injury (TBI) is a major global cause of mortality and long‐term disability, with limited therapeutic options. Microglial pyroptosis, a form of programmed cell death associated with inflammation, has been implicated in exacerbating neuroinflammation and secondary injury following TBI. Compound porcine cerebroside ganglioside injection (CPCGI) has shown anti‐inflammatory and antioxidant properties, but its effects on pyroptosis remain unexplored. This study investigates the role of CPCGI in TBI and its underlying mechanisms.

**Methods:**

A controlled cortical impact (CCI) model was utilized to establish TBI in vivo, while lipopolysaccharide (LPS) was used in vitro to induce microglial activation that mimicked TBI conditions. The effects of CPCGI on microglial pyroptosis and inflammatory cytokines were analyzed through immunofluorescence, flow cytometry, western blotting, and quantitative real‐time PCR (qRT‐PCR). The involvement of the NLRP3 inflammasome in CPCGI's mechanism was examined using NLRP3 overexpression or the NLRP3 agonist BMS‐986299. A microglia–neuron interaction model was created, and neuronal injury was assessed with the Cell Counting Kit‐8 and Fluoro‐Jade C (FJC).

**Results:**

Treatment with CPCGI resulted in significant improvement in the neurobehavioral outcomes, reduced lesion volume, and decreased neuronal loss following TBI. Notably, TBI induced microglial pyroptosis and the release of pro‐inflammatory cytokines, while CPCGI inhibited microglial pyroptosis, thereby mitigating the inflammatory response and reducing neuronal damage. Mechanistically, overexpression of NLRP3 in microglial cells reversed the inhibitory effects of CPCGI on microglial pyroptosis, indicating that CPCGI's inhibition of microglial pyroptosis may be mediated by the NLRP3 inflammasome. Furthermore, NLRP3 overexpression or administration of the NLRP3 agonist BMS‐986299 negated the neuroprotective effects of CPCGI in vivo and in vitro.

**Conclusion:**

These findings suggest that CPCGI provides neuroprotection in TBI by targeting NLRP3 inflammasome‐mediated microglial pyroptosis, thereby improving the neuroinflammatory microenvironment and promoting neurological recovery. This underscores its potential as a promising candidate for TBI treatment.

## Graphical abstract

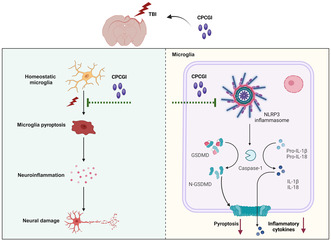

## 1 Introduction

Traumatic brain injury (TBI) encompasses structural and functional disruptions in brain tissue caused by external forces and is a major contributor to global mortality and disability [1, 2, 3, 4]. In China, the incidence of TBI is among the highest worldwide, positioning it as a critical public health issue [5]. TBI has increasingly been recognized not only as an acute event but also as a chronic condition with enduring repercussions, including a heightened risk of late‐onset neurodegenerative disorders [6]. Despite ongoing research, effective therapeutic strategies remain limited, often failing to enhance patient outcomes, which leads to significant rates of mortality and long‐term disability. A pivotal secondary injury mechanism in TBI is neuroinflammation, which can lead to neuronal cell death, compromise the blood–brain barrier, and exacerbate cerebral edema [7, 8, 9, 10, 11, 12, 13]. Therefore, strategies aimed at mitigating neuroinflammation are crucial for alleviating secondary damage and promoting recovery in TBI patients.

Pyroptosis is an inflammatory form of programmed cell death that distinctly differs from necrosis and apoptosis [14, 15]. This process is characterized by cellular swelling, the formation of pores in the plasma membrane, and subsequent membrane rupture, which results in the release of inflammatory cytokines and a pronounced inflammatory response [14, 16, 17]. Following TBI, microglia—the resident immune cells of the central nervous system—can be rapidly activated, initiating a cascade of neuroinflammatory events [18]. A critical component of this response is the activation of the NLR family pyrin domain‐containing protein 3 (NLRP3) inflammasome in microglia [8, 9, 19, 20]. The NLRP3 inflammasome, a multiprotein complex composed of NLRP3, apoptosis‐associated speck‐like protein containing a CARD (ASC), and pro‐caspase‐1, is widely distributed in both immune and non‐immune cells and plays a pivotal role in mediating inflammatory responses [21, 22, 23, 24, 25]. Gasdermin D (GSDMD) acts as a key effector of the NLRP3 inflammasome; upon activation of pro‐caspase‐1, GSDMD is cleaved, generating N‐terminal fragments that oligomerize and form pyroptosis pores in the cell membrane. This process facilitates the release of inflammatory cytokines, such as IL‐1β and IL‐18, thereby triggering pyroptosis [23, 26, 27]. Recent research has identified pyroptosis as a significant contributor to post‐TBI inflammation, underscoring its importance in the pathophysiology of TBI [9]. Thus, targeting pyroptosis presents a promising therapeutic avenue for TBI.

Compound porcine cerebroside ganglioside injection (CPCGI) is a composite formulation that includes 3.2 mg of peptide, 0.24 mg of monosialotetrahexosyl ganglioside (GM‐1), and 0.125 mg of hypoxanthine per ml [28]. Clinically, it is employed to treat acute and chronic cerebrovascular diseases and brain dysfunction resulting from trauma [29, 30, 31, 32]. Research indicates that CPCGI exhibits anti‐apoptotic, antioxidant, and anti‐inflammatory properties in the context of cerebral ischemia–reperfusion injury [33, 34]. However, the potential of CPCGI to mitigate neuronal damage in TBI by inhibiting microglial pyroptosis and modulating inflammatory responses remains inadequately explored. This study aims to elucidate the role of microglial pyroptosis‐induced neuroinflammation in the neurological damage associated with TBI and to determine whether CPCGI exerts neuroprotective effects through the inhibition of this pathway.

## 2 Methods

### 2.1 Animals and Models of TBI

Male C57BL/6 mice, aged 8–10 weeks and weighing between 20 and 24 g, were sourced from SPF (Beijing) Biotechnology Co. Ltd. The mice were housed under strictly regulated environmental conditions, maintaining a 12‐h light/dark cycle, with humidity levels set at 55% ± 5% and a temperature of 22°C ± 2°C. They had unrestricted access to food and water. Prior to the initiation of experimental procedures, the animals were allowed to acclimate to their surroundings for at least three days. All experimental protocols were conducted in accordance with NIH guidelines for the care and use of laboratory animals and adhered to the ARRIVE guidelines for reporting research findings [35, 36].

The controlled cortical impact (CCI) model was employed to induce traumatic brain injury. Anesthesia was achieved using 3%–5% isoflurane, and the mice were secured in a stereotaxic apparatus, with maintenance anesthesia provided at 1.5% isoflurane via a nose cone throughout the surgical procedure. A 4‐mm diameter craniotomy was performed at coordinates anteroposterior (AP) − 2.0 mm and mediolateral (ML) + 2.0 mm, ensuring that the dura mater remained intact. Cortical contusion was induced using controlled cortical impact equipment (Leica 9969S) fitted with a 3‐mm diameter impactor tip. The impact velocity was set at 3.0 m/s, with a contact duration of 100 ms. Based on previous experimental findings, the moderate injury model was identified as optimal, characterized by an impact depth of 1.0 mm [37, 38, 39]. Following the TBI procedure, the mice were placed on a heating pad to facilitate recovery. Mice in the sham group underwent the same surgical preparation but did not experience CCI injury.

### 2.2 Drug Administration and Experimental Design

**FIGURE 1:**
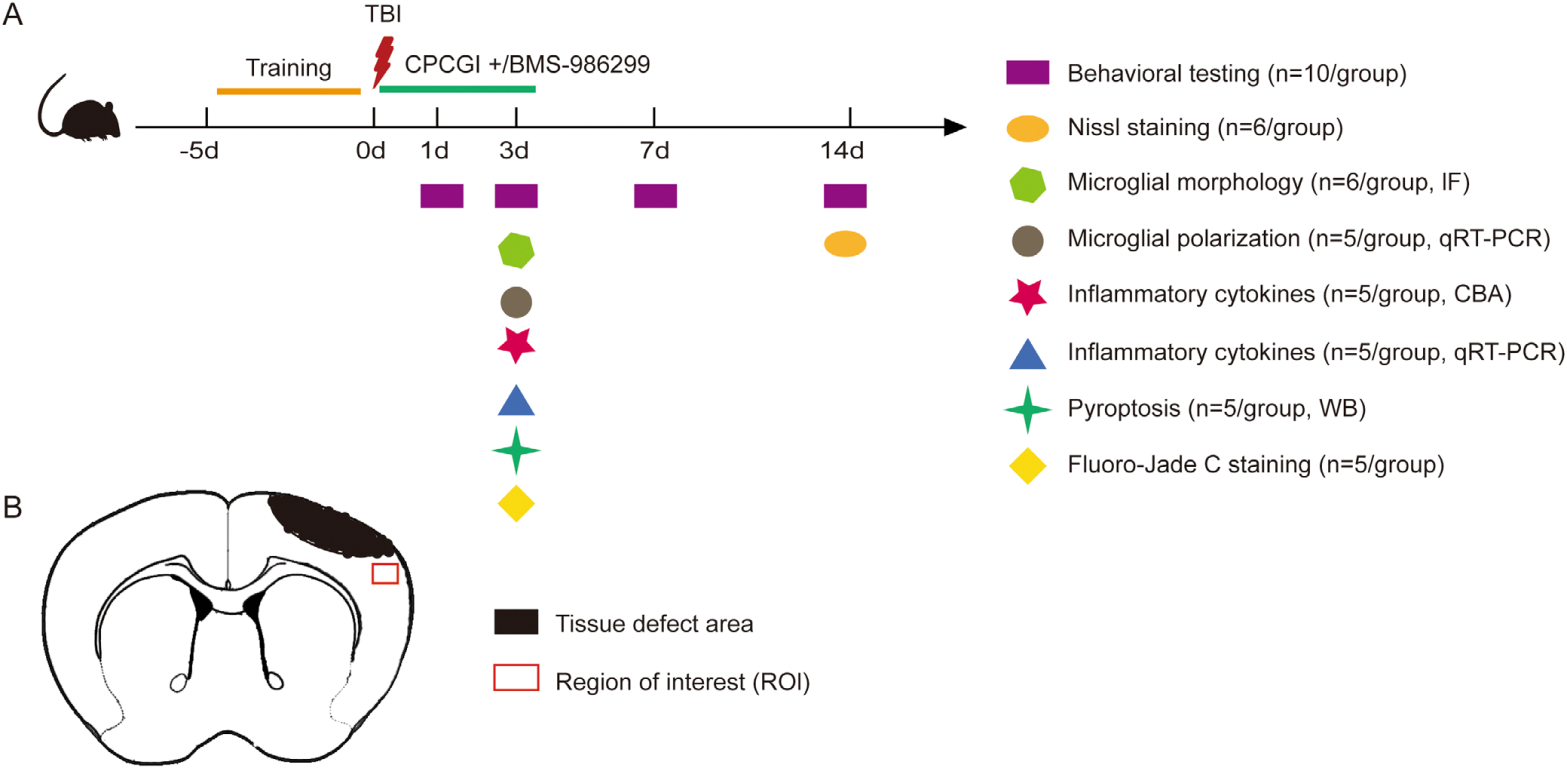
Experimental outline and schematic diagram of brain section. (A) Overview of experimental timeline for in vivo studies and neurobehavioral tests. (B) Schematic diagram of brain section. Red squares indicate the region of interest in the ipsilateral cortex, in which immunofluorescence images were collected.

CPCGI, produced by Buchang Pharmaceutical Group Ltd. in Jilin, China, received approval from the China Food and Drug Administration in 2010. The allocation of mice into experimental groups was conducted randomly using a number table generated by SPSS software version 21.0. In the initial experiments, the mice were divided into four distinct groups: the TBI + vehicle group (which underwent controlled cortical impact and was administered an equivalent volume of 0.9% saline), the TBI + CPCGI‐L group (which received intraperitoneal CPCGI at a dosage of 0.5 mL/kg/day), the TBI + CPCGI‐M group (administered CPCGI at 1 mL/kg/day), and the TBI + CPCGI‐H group (administered CPCGI at 2 mL/kg/day). In the subsequent studies, the mice were categorized into three groups: the sham group (which underwent a sham procedure and received an equivalent volume of 0.9% saline), the TBI + vehicle group, and the TBI + CPCGI group (which underwent a TBI procedure and receiving CPCGI at a dosage of 1 mL/kg/day). BMS‐986299 (10 mg/kg/day, ip. MedChemExpress, #HY‐139396) is a specific agonist of the NLRP3 inflammasome. Finally, the mice were divided into five distinct groups: sham group, TBI + vehicle group, TBI + CPCGI group, TBI + BMS group (which underwent a TBI procedure and receiving BMS‐986299), and TBI + CPCGI + BMS group (which underwent a TBI procedure and receiving CPCGI and BMS‐986299). At 3 and 14 days post‐TBI, in accordance with the experimental protocol, the mice were euthanized through rapid decapitation under deep anesthesia, and tissue samples were collected for subsequent analysis. The experimental outline is shown in Figure 1A.

### 2.3 Neurobehavioral Tests

Neurobehavioral assessments were performed by an investigator who remained unaware of the treatment groups. Evaluations were conducted prior to the TBI and continued for 14 days post‐injury. Sensorimotor deficits were evaluated using the rotarod test, modified neurological severity score (mNSS), adhesive removal test and corner test [40, 41].

#### 2.3.1 Rotarod Test

The rotarod apparatus was employed to assess the motor learning and coordination capabilities of the mice. Prior to the TBI, subjects participated in a five‐day training protocol. Each mouse was placed on a rotating rod set at a fixed speed of 4 rpm, with three trials conducted daily and 15min intervals between each trial. Only those mice that successfully remained on the rod for at least 60 s were included in the analysis. The assessment involved placing the mice on a rod that accelerated from 4 to 40 rpm over a four‐minute period, with each mouse undergoing three trials. The final score was determined as the average duration that each mouse maintained balance on the rod across all trials.

#### 2.3.2 mNSS

The mNSS provides a comprehensive evaluation of motor, sensory, reflexive, and balance functions, rated on a scale from 0 to 18, where 0 indicates normal function and 18 denotes maximum impairment. Subjects receive one point for each absent reflex or task they are unable to perform, with higher scores reflecting greater neurological deficits.

#### 2.3.3 Adhesive Removal Test

In this test, a 3 mm × 3 mm piece of adhesive tape was attached to the palmar surface of the contralateral forepaw. The time taken for the mouse to contact and subsequently remove the adhesive was recorded. Each subject underwent three trials daily, beginning three days prior to the TBI and continuing until specified postoperative time points. The average performance from the three trials conducted the day before surgery was utilized to establish the baseline.

#### 2.3.4 Corner Test

The corner test was employed to evaluate sensorimotor and postural asymmetries following TBI. The apparatus consists of two boards (30 × 20 × 1 cm^3^) positioned at a 30° angle, creating a small opening to encourage the mouse to enter the corner. The mouse was gently held by the tail and placed between the boards, facing the open end and positioned halfway toward the corner. A total of ten trials were conducted, recording the number of left and right turns. The asymmetry score was calculated as follows:

$$\text{Asymmetry}\;\text{score}=\left(\frac{\text{Number}\;\text{of}\;“\text{standing}\;\text{turns}”\;\text{to}\;\text{the}\;\text{injury}\;\text{side}}{\text{Total}\;\text{number}\;\text{of}\;“\text{standing}\;\text{turns}”}\right)\times 100\%$$

A higher score indicates a more severe lesion.

### 2.4 Nissl Staining

Nissl staining was conducted following established protocols [17]. Brain tissue sections at the desired level were selected and left at room temperature for minutes to rewarm. The sections were washed three times with double‐distilled water (ddH_2_O) for 5 min each time. Staining was performed in a fume hood for 5–10 min, followed by dehydration in 70% alcohol I, 70% alcohol II, 95% alcohol I, 95% alcohol II, 100% alcohol I, and 100% alcohol II for a few seconds to a few minutes and xylene clearing twice for 2 min each time. The slices were sealed with neutral gum, dried, and photographed. Images were captured using a Phenolmager multiplexed imaging system (Akoya Biosciences). The damage volume was calculated by the formula: [(*V*_*C*_ − *V*_*L*_)/*V*_*C*_] × 100% (*V*_*C*_: volume of the contralateral hemisphere; *V*_*L*_: volume of the ipsilateral hemisphere). The number of neurons in the region of interest (ROI) in the ipsilateral cortex was analyzed with ImageJ software (Figure 1B).

### 2.5 Immunofluorescence Staining

TBI mice were deeply anesthetized via intraperitoneal injection of Avertin (400 mg/kg; Sigma‐Aldrich, #T48402‐25G) and transcardially perfused with 0.9% NaCl followed by 4% paraformaldehyde (PFA). Coronal brain sections (15 μm thick) were prepared using a cryostat (Thermo Scientific, USA). Sections were permeabilized with 0.3% Triton X‐100 for 15 min, blocked with 10% normal donkey serum at 37°C for 1 h, and incubated overnight at 4°C with primary antibodies: goat anti‐Iba1 (1:200; Abcam, #ab289874). The following day, sections were washed with PBS and incubated with secondary antibodies (Alexa Fluor 488, Jackson ImmunoResearch, USA) at 37°C for 1 h. Control sections were processed in parallel without primary antibodies. Fluorescent images were captured using a Zeiss LSM880 laser scanning confocal microscope (Germany). 3D reconstruction was performed using Imaris software. Quantification of Iba1+ cells, microglial endpoints per cell, and process length per cell was performed using ImageJ software.

### 2.6 Cytometric Bead Array

Cytokine concentrations of IL‐1β, IL‐18, IL‐6, and TNF‐α in mouse serum or BV2 cell supernatant were quantified using the BD Cytometric Bead Array Mouse/Rat Soluble Protein Master Buffer Kit (Becton, Dickinson and Company, USA). The protocol for preparing standards, capture beads, detection reagents, and samples, as well as flow cytometer setup and data analysis, was strictly followed according to the manufacturer's instructions. In brief, standards were reconstituted and serially diluted immediately before use. Fifty microliters of prepared capture beads were added to each assay tube, followed by 50 μL of either standards or samples. The tubes were thoroughly mixed and incubated at room temperature for 1 h in the dark. Next, 50 μL of PE detection reagent was added to each tube, with an additional 1h incubation at room temperature. Samples were then washed with 1.0 mL of wash buffer and centrifuged at 200 *g* for 5 min, and the supernatant was discarded. Beads were resuspended in 300 μL of wash buffer. Data acquisition was performed using a BD FACS Aria II flow cytometer, with data analyzed using FCAP Array software (v3.0, BD Biosciences).

### 2.7 Quantitative Real‐Time PCR (qRT‐PCR)

The total RNA was extracted from brain tissues and BV2 cells using the Trizol reagent (Invitrogen, CA) according to the manufacturer's protocol. Complementary DNA (cDNA) was synthesized using the PrimeScript First Strand cDNA Synthesis Kit (Takara, Japan). PCR was performed with PowerUp SYBR Green Master Mix (Thermo Fisher Scientific, USA) on a LightCycler 480 PCR System (Roche, USA). Primer pairs were designed using the NCBI Primer‐BLAST tool (http://www.ncbi.nlm.nih.gov/tools/primer‐blast/) to minimize the risk of non‐specific amplification. All primers demonstrated efficiencies between 90% and 105%. Gene expression levels were normalized to *GAPDH*, and relative expression was calculated using the 2^−ΔΔCt^ method. Primer sequences for target genes are detailed in Table 1.

**TABLE 1:** Mouse primer sequences.

Gene	Forward primer (5′‐3′)	Reverse primer (5′‐3′)
*NLRP3*	CCAGCCAGAGTGGAATGACA	AGCGGGAGACAAATGGAGAT
*NLRP1a*	GCCTCACATCCACATACTGCTCAC	CTGACTGCTGTCTCTGCTGCTTC
*NLRC4*	GCGAGTCTGGCAAAGGGAAGTC	CCGTGGTGGTGGTGACAATGAC
*AIM2*	CTGTCTGCCGCCATGCTTCC	ACTGTCTTGTTCCCACTGCCTTTG
*IL‐1β*	AACTCAACTGTGAAATGCCACC	CATCAGGACAGCCCAGGTC
*IL‐18*	GACAGCCTGTGTTCGAGGATATG	TGTTCTTACAGGAGAGGGTAGAC
*IL‐6*	CACTTCACAAGTCGGAGGCT	CTGCAAGTGCATCATCGTTGT
*TNF‐a*	GGTGCCTATGTCTCAGCCTCTT	GCCATAGAACTGATGAGAGGGAG
*CD16*	TTTGGACACCCAGATGTTTCAG	GTCTTCCTTGAGCACCTGGATC
*CD206*	TCTTTGCTTTCCAGTCTCC	TGACACCCAGCGGAATTTC
*TGF‐α*	TTCTCATTCCTGCTTGTGG	ACTTGGTGGTTTGCTACG
*IL‐10*	TGCCTTCAGTCAAGTGAAGAC	AAACTCATTCATGGCCTTGTA
*Arg‐1*	TCCTTAGAGATTATCGGAGCG	GTCTTTGGCAGATATGCAGG
*YM‐1*	CAGGGTAATGAGTGGGTTGG	CACGGCACCTCCTAAATTGT
*GAPDH*	TTGATGGCAACAATCTCCAC	CGTCCCGTAGACAAAATGGT

### 2.8 Western Blot

Proteins from brain tissue and cultured cells were extracted using radio immunoprecipitation assay (RIPA) buffer (Solarbio, #R0020) supplemented with 1% protease inhibitor cocktail (Sigma, #P8340) and 1% phosphatase inhibitor (Applygen, #P1260) at 4°C. Protein concentrations were quantified using the bicinchoninic acid (BCA) protein assay kit (Thermo Scientific, USA). For each sample, 30 μg of protein was separated by 10% SDS‐PAGE and transferred onto PVDF membranes (Roche, USA). Membranes were blocked with 5% nonfat milk in TBST at room temperature for 1 h, followed by overnight incubation at 4°C with primary antibodies: NLRP3 (1:1000, Abmart, #P60622R3S), pro‐Caspase1 (1:1000, AdipoGen, #AG‐20B‐0042), p20 Caspase1 (1:1000, AdipoGen, #AG‐20B‐0042), and GSDMD (1:1000, Abcam #ab219800). GAPDH (1:1000, Bioworld Technology, #AP0063) was used as a loading control. After primary antibody incubation, membranes were washed three times with TBST and incubated for 1 h at room temperature with a secondary antibody, Goat Anti‐Rabbit IgG (H&L) DyLight 800 conjugated (1:10,000, Rockland, #611‐145‐122). Protein bands were detected using the Odyssey infrared imaging system (LICOR Biosciences, USA). Band intensities were analyzed with ImageJ software, and the relative expression levels of NLRP3, pro‐Caspase1, p20 Caspase1, and GSDMD were normalized to GAPDH in both in vivo and in vitro samples.

### 2.9 Cell Culture and Treatments

Microglial BV2 cell lines were obtained from the China Center for Type Culture Collection and maintained in DMEM (Gibco, USA) supplemented with 10% fetal bovine serum (FBS; Gibco, USA) and 1% penicillin–streptomycin (Solarbio, China). Cells were cultured at 37°C in a humidified incubator with 5% CO_2_ and passaged every 48 h. After 24 h of subculture, BV2 cells were treated with LPS (1 μg/mL) and varying concentrations of CPCGI (0, 1, 2, 5, and 10 μM) for 24 h, except in the Control group. BV2 cells were then divided into the following groups: Control (no LPS or CPCGI treatment), LPS (LPS‐only treatment), and LPS + CPCGI (LPS + 5 μM CPCGI treatment). Next, a lentiviral vector containing NLRP3 (NM_001243133.2) was constructed to overexpress the NLRP3 gene (OE‐NLRP3, BrainCase, China). BV2 cells were transduced with either OE‐NLRP3 or a control lentivirus expressing GFP (OE‐NC) at a multiplicity of infection (MOI) of 5. BV2 cells were divided into 5 groups: Control, OE‐NC LPS (BV2 cells were transduced with OE‐NC and treated with LPS), OE‐NC LPS + CPCGI (BV2 cells were transduced with OE‐NC and treated with LPS and CPCGI), OE‐NLRP3 LPS (BV2 cells were transduced with OE‐NLRP3 and treated with LPS), and OE‐NLRP3 LPS + CPCGI (BV2 cells were transduced with OE‐ NLRP3 and treated with LPS and CPCGI). Cell viability and cytometric bead array assays were used to assess cellular responses. Each group's BV2 cell supernatant was gathered to create conditioned medium (CM), which was then administered to primary neurons.

Primary cortical neurons were isolated from the brains of C57BL/6 mice embryos at E15‐18. Following established protocols with minor modifications, cerebral cortices were dissected, dissociated in isotonic buffer, and digested with 2.0 mg/mL papain (Solarbio, China) and DNAse (Solarbio, China) at 37°C for 20 min. Dissociated cells were plated at 7 × 10^5^ cells/well on poly‐L‐lysine‐coated 6‐well plates (Corning USA) and cultured in neurobasal medium with 2% B27 supplement and 0.5 mM glutamine (Gibco, USA) at 37°C in a 5% CO_2_ incubator. On day 7, neurons were immunostained with anti‐MAP2 to confirm neuronal identity. On day 7, primary neurons were divided into four treatment groups: Control, CM (Control), CM (LPS), and CM (LPS + CPCGI). Next, primary neuron cells were divided into 5 groups: CM (Control), CM (OE‐NC LPS), CM (OE‐NC LPS + CPCGI), CM (OE‐NLRP3 LPS), and CM (OE‐NLRP3 LPS + CPCGI). For CM groups, half of the medium was replaced with filtered CM from the BV2 cell supernatant [41]. Prior to application, CM was centrifuged at 200 g for 5 min and filtered through a 0.22 μm membrane to remove residual compounds. Cell viability assay was used to assess cellular responses.

### 2.10 Cell Viability Assay

Cell viability was assessed using the Maximum Sensitivity Cell Counting Kit‐8 (CCK‐8, Abbkine, China). Cells were seeded in 96‐well plates at a density of 1 × 10^5^ cells/mL. Following CPCGI treatment, 10 μL of CCK‐8 solution was added to each well, and the plates were incubated at 37°C for 2 h. Absorbance at 450 nm was measured using a microplate reader (TECAN). Viability for each treatment group was expressed as a percentage relative to the control group. Each group was tested in quintuplicate, and results are representative of three independent experiments [40].

### 2.11 Fluoro‐Jade C (FJC) Staining

Degenerating neurons were identified using Fluoro‐Jade C staining, with the modified FJC Ready‐to‐Dilute Staining Kit (Biosensis, AUS), performed 3 days post‐TBI as previously described [42]. According to the manufacturer's instructions, brain tissue sections were incubated in a mixture of 80% ethanol and sodium hydroxide for 5 min, then transferred to 70% ethanol for 2 min, and rinsed in distilled water for 2 min. Next, sections were incubated in potassium permanganate for 10 min, followed by 10 min in Fluoro‐Jade C (FJC) solution in the dark. After rinsing three times in distilled water for 1 min each, slides were dried on a slide warmer at 50°C–60°C. Finally, the dried slides were briefly immersed in xylene for 1–5 min for clearing before mounting with a coverslip. Neurons exhibiting FJC positivity were visualized with a Zeiss LSM880 laser scanning confocal microscope (Zeiss, Germany) and manually counted in the cortex for each brain section using ImageJ software. The results are expressed as the average number of FJC‐positive cells per field. Sections from each brain were obtained and analyzed by two researchers blinded to group assignments. All FJC‐positive cells were counted across sections for each brain in a blinded manner using fluorescence microscopy.

### 2.12 Statistical Analysis

Data are presented as mean ± SD. Statistical approaches for metabolomics and proteomics analyses are specified in their respective sections. For data following a normal distribution, parametric tests were employed: comparisons between two groups used the two‐tailed Student's *t*‐test, while comparisons among three or more groups were analyzed with one‐way ANOVA, followed by the least significant difference (LSD) test for equal variances or Dunnett's test for unequal variances. Non‐normally distributed data were analyzed using the Kruskal–Wallis test with Dunn's post hoc test for multiple comparisons. Sample sizes were calculated by the ‘resource equation’ method [43]. To control Type I error across multiple comparisons, adjustments were made using LSD, Dunnett's, Dunn's, and Bonferroni's methods. Statistical significance was defined as *p* < 0.05. All statistical analyses and visualizations were conducted using SPSS software (version 21.0; SPSS Inc., Chicago, IL), and curve fitting was performed with GraphPad Prism (version 8.0.2; GraphPad Software).

## 3 Results

### 3.1 CPCGI Improved Neurological Recovery and Attenuated Cerebral Lesion Volume After TBI

**FIGURE 2:**
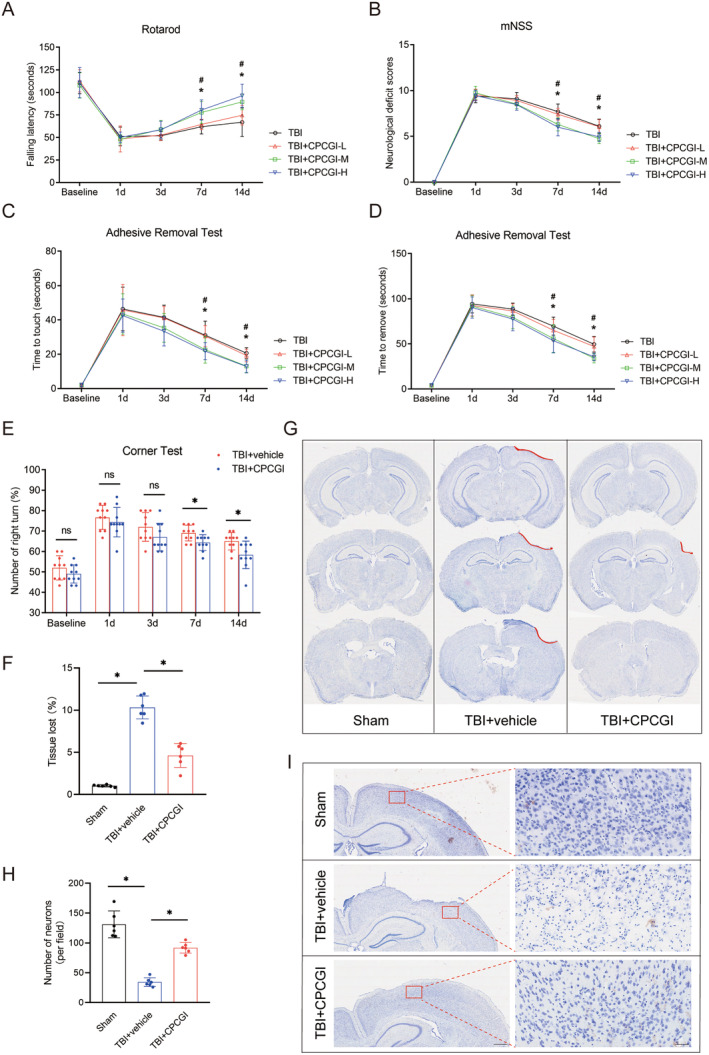
CPCGI improved neurological recovery and attenuated cerebral lesion volume in TBI mice. (A) Rotarod test. *p = 0.000783 at 7 days, and *p = 0.000238 at 14 days (TBI + CPCGI‐M vs. TBI); #*p* = 0.000124 at 7 days, and #*p* = 0.000006 at 14 days (TBI + CPCGI‐H vs. TBI); one‐way ANOVA followed by LSD multiple comparison tests. *p* = 0.000003 by repeated‐measures ANOVA. *n* = 10. (B) mNSS. *p = 0.000348 at 7 days, and *p = 0.02 at 14 days (TBI + CPCGI‐M vs. TBI); #*p* = 0.000028 at 7 days, and #*p* = 0.012 at 14 days (TBI + CPCGI‐H vs. TBI); one‐way ANOVA followed by LSD multiple comparison tests at the 7 days time points. The Kruskal–Wallis test followed by Dunn's post hoc analysis was performed at the 14 days time points. *p* = 0.000004 by repeated‐measures ANOVA. *n* = 10. (C) Time to touch the adhesive tape. *p = 0.009887 at 7 days, and *p = 0.000032 at 14 days (TBI + CPCGI‐M vs. TBI); ^#^ *p* = 0.005012 at 7 days, and ^#^ *p* = 0.000023 at 14 days (TBI + CPCGI‐H vs. TBI); one‐way ANOVA followed by LSD multiple comparison tests. *p* = 0.000218 by repeated‐measures ANOVA. *n* = 10. (D) Time to remove the adhesive tape. *p = 0.022492 at 7 days, and *p = 0.000045 at 14 days (TBI + CPCGI‐M vs. TBI); ^#^ *p* = 0.008214 at 7 days, and ^#^ *p* = 0.000178 at 14 days (TBI + CPCGI‐H vs. TBI); one‐way ANOVA followed by LSD multiple comparison tests. *p* = 0.000013 by repeated‐measures ANOVA. *n* = 10. (E) Corner test. *p = 0.014657 at 7 days, and *p = 0.016051 at 14 days (TBI + CPCGI vs. TBI + vehicle); Student's t test at individual time points. (F and G) Tissue loss were investigated using Nissl staining and quantification of the tissue loss at 14 days after TBI in the sham, TBI + vehicle and TBI + CPCGI groups. *p* = 0.000034 (TBI + vehicle vs. sham); *p* = 0.000101 (TBI + CPCGI vs. TBI + vehicle); one‐way ANOVA followed by Dunnett's multiple comparison tests, *n* = 6. (H and I) Number of neurons were investigated using Nissl staining and quantification of the number of neurons at 14 days after TBI in the sham, TBI + vehicle and TBI + CPCGI groups. *p* = 0.000150 (TBI + vehicle vs. sham); *p* = 0.000001 (TBI + CPCGI vs. TBI + vehicle); one‐way ANOVA followed by Dunnett's multiple comparison tests, *n* = 6. Left figures bar = 500 μm, right figures bar = 50 μm.

To assess the efficacy of CPCGI in mitigating traumatic brain injury, we evaluated neurological function on days 1, 3, 7, and 14 post‐TBI using the rotarod test, modified neurological severity score (mNSS), and adhesive removal test. Mice treated with a moderate dose of CPCGI (CPCGI‐M) showed significant improvement in the rotarod test from day 7 to day 14 compared with untreated TBI mice, indicating enhanced motor coordination and learning ability (day 7, 77.77 ± 12.04 vs. 62.00 ± 8.09; day 14, 89.47 ± 8.80 vs. 67.00 ± 15.70; *p* < 0.05, Figure 2A). High‐dose CPCGI (CPCGI‐H) also significantly improved performance on the rotarod at 7 and 14 days post‐TBI (day 7, 80.49 ± 11.21 vs. 62.00 ± 8.09; day 14, 96.23 ± 12.85 vs. 67.00 ± 15.70; *p* < 0.05, Figure 2A). Further, mNSS scores showed marked improvements in both the TBI + CPCGI‐M and TBI + CPCGI‐H groups compared to the TBI + vehicle group on days 7 and 14 (TBI + CPCGI‐M vs. TBI: 7 days, 6.30 ± 0.71 vs. 7.70 ± 0.82; 14 days, 4.75 ± 0.54 vs. 6.10 ± 0.77. TBI + CPCGI‐H vs. TBI: 7 days, 6.00 ± 0.94 vs. 7.70 ± 0.82; 14 days, 4.95 ± 0.50 vs. 6.10 ± 0.77. *p* < 0.05, Figure 2B). Consistent with these findings, CPCGI‐M and CPCGI‐H treated mice exhibited quicker response times to touch and adhesive removal from the forepaw compared to untreated TBI mice, indicating that CPCGI mitigated sensory‐motor deficits post‐TBI (*p* < 0.05, Figure 2C,D). Based on the observed therapeutic benefits at doses of 1 and 2 mL/kg/day, a dose of 1 mL/kg/day was selected for subsequent in vivo studies.

To better reflect damage to the barrel cortex, we performed the corner test. As expected, both the TBI + vehicle and TBI + CPCGI groups exhibited behavioral asymmetries on the corner test following right‐sided CCI. However, animals in the TBI + CPCGI group showed a significant reduction in the number of right turns compared to the TBI + vehicle group at both 7 and 14 days post‐injury (TBI + CPCGI vs. TBI + vehicle: 7 days, 64.33 ± 3.87 vs. 69.00 ± 3.86; 14 days, 58.33 ± 6.71 vs. 65.00 ± 4.23; *p* < 0.05, Figure 2E), indicating that CPCGI treatment mitigated some of the barrel cortex dysfunction typically observed after TBI.

Nissl staining was conducted on brain sections from TBI and age‐matched sham mice to assess cerebral lesion volume. Analysis revealed a significant increase in the cerebral lesion volume in the TBI + vehicle group compared to the sham group at 14 days post‐TBI, particularly in the ipsilateral cortex (TBI + vehicle vs. sham: 10.32 ± 1.36 vs. 1.03 ± 0.13; *p* < 0.05, Figure 2F,G). The sham group exhibited no gross cerebral lesions. Notably, CPCGI treatment significantly reduced cerebral lesion volume relative to the TBI + vehicle group (TBI + CPCGI vs. TBI + vehicle: 4.63 ± 1.42 vs. 10.32 ± 1.36; *p* < 0.05, Figure 2F,G). In the TBI + vehicle group, stereological analysis of Nissl‐stained sections revealed a significantly reduced neuronal count in the peri‐injury cortex compared to the sham group (34.27 ± 7.33 vs. 131.06 ± 22.44, *p* < 0.05, Figure 2H,I). Treatment with CPCGI significantly improved neuronal survival at 14 days post‐TBI, as evidenced by a higher neuron count in CPCGI‐treated mice compared to the TBI + vehicle group (91.78 ± 8.85 vs. 34.27 ± 7.33, *p* < 0.05, Figure 2H,I).

### 3.2 CPCGI Ameliorated Microglia‐Mediated Neuroinflammation After TBI

**FIGURE 3:**
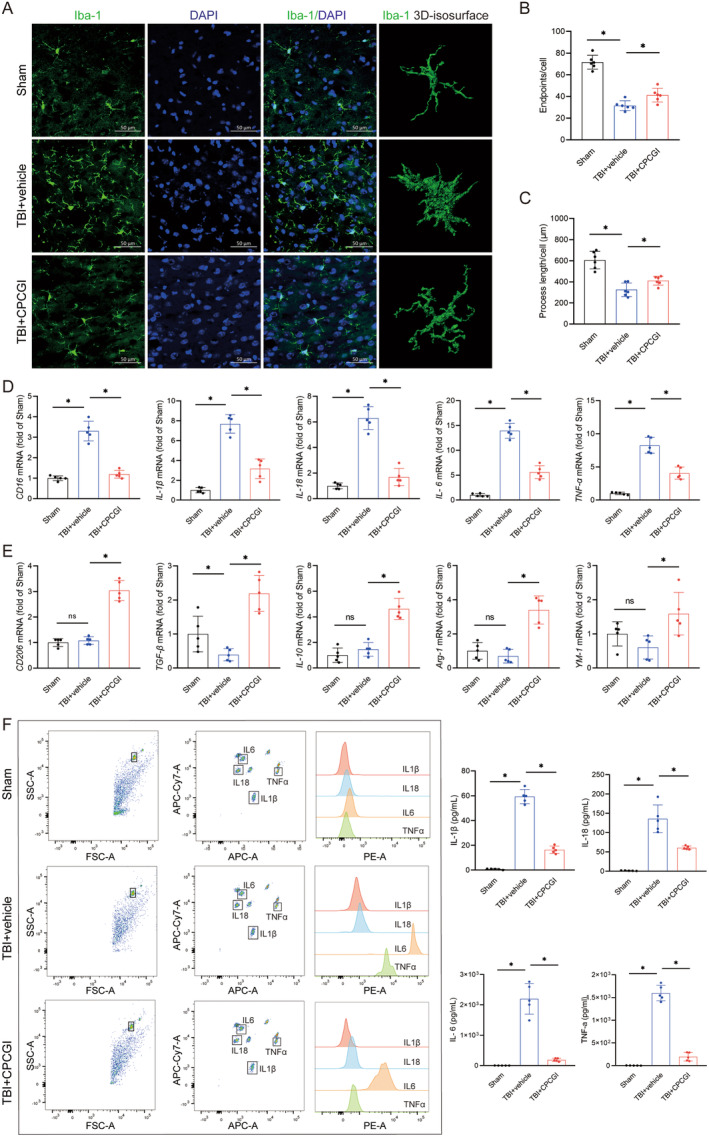
CPCGI ameliorated microglia‐mediated neuroinflammation after TBI. (A) Representative images of microglia at 3 days after TBI, and examples of Imaris 3D cellular isosurface rendering to illustrate microglial morphology. (B and C) Quantification of microglial endpoints/cell (B) and process length/cell (C); Microglial endpoints/cell. *p* < 0.001 (TBI + vehicle vs. sham); *p* = 0.011906 (TBI + CPCGI vs. TBI + vehicle); Microglial process length/cell. *p* = 0.000002 (TBI + vehicle vs. sham); *p* = 0.037832 (TBI + CPCGI vs. TBI + vehicle); one‐way ANOVA followed by LSD multiple comparison tests. *n* = 6. (D) The mRNA expression of pro‐inflammatory markers (CD16, IL‐1β, IL‐18, IL‐6, and TNF‐α) of microglia at 3 days after TBI. CD16: *P* < 0.001 (TBI + vehicle vs. Sham), *p* < 0.001 (TBI + CPCGI vs. TBI + vehicle); IL‐1β, *p* = 0.000103 (TBI + vehicle vs. Sham), *p* = 0.000223 (TBI + CPCGI vs. TBI + vehicle); IL‐18, *p* < 0.001 (TBI + vehicle vs. Sham), *p* < 0.001 (TBI + CPCGI vs. TBI + vehicle); IL‐6, *p* < 0.001 (TBI + vehicle vs. Sham), *p* < 0.001 (TBI + CPCGI vs. TBI + vehicle); TNF‐α, *p* = 0.000351 (TBI + vehicle vs. Sham), *p* = 0.000952 (TBI + CPCGI vs. TBI + vehicle); one‐way ANOVA followed by Dunnett's multiple comparison tests (IL‐1β and TNF‐α) or LSD multiple comparison tests (CD16, IL‐18 and IL‐6). *n* = 5. (E) The mRNA expression of anti‐inflammatory markers (CD206, TGF‐β, IL‐10, Arg‐1 and YM‐1) of microglia at 3 days after TBI. CD206: *p* = 0.000325 (TBI + CPCGI vs. TBI + vehicle); TGF‐β, *p* = 0.049102 (TBI + vehicle vs. Sham), *p* = 0.000031 (TBI + CPCGI vs. TBI + vehicle); IL‐10, *p* = 0.000006 (TBI + CPCGI vs. TBI + vehicle); Arg‐1, *p* = 0.001839 (TBI + CPCGI vs. TBI + vehicle); YM‐1, *p* = 0.005214 (TBI + CPCGI vs. TBI + vehicle); one‐way ANOVA followed by Dunnett's multiple comparison tests (CD206 and Arg‐1) or LSD multiple comparison tests (TGF‐β, IL‐10 and YM‐1). *n* = 5. (F) The concentrations of IL‐1β, IL‐18, IL‐6, and TNF‐α in the plasma samples from mice at 3 days after TBI. IL‐1β, *p* < 0.001 (TBI + vehicle vs. Sham), *p* < 0.001 (TBI + CPCGI vs. TBI + vehicle). IL‐18, *p* = 0.002896 (TBI + vehicle vs. Sham), *p* = 0.023571 (TBI + CPCGI vs. TBI + vehicle). IL‐6, *p* = 0.001619 (TBI + vehicle vs. Sham), *p* = 0.002122 (TBI + CPCGI vs. TBI + vehicle). TNF‐α, *p* = 0.000080 (TBI + vehicle vs. Sham), *p* = 0.000008 (TBI + CPCGI vs. TBI + vehicle). One‐way ANOVA followed by LSD multiple comparison tests (IL‐1β) or Dunnett's multiple comparison tests (IL‐18, IL‐6, and TNF‐α). *n* = 5.

Microglia, the primary immune cells in the central nervous system, play a critical role in initiating inflammatory responses after traumatic brain injury. Activated microglia rapidly migrate to the injury site and transition between pro‐inflammatory and anti‐inflammatory phenotypes, both of which are central to the neuroinflammatory response. Recent studies highlight that modulating microglial polarization, particularly promoting the anti‐inflammatory phenotype, could serve as a therapeutic strategy in TBI management [8, 19, 20, 44]. To assess the effects of CPCGI on microglial activity, immunofluorescence staining was used to analyze microglial morphology (Figure 3A and Figure S1A). Microglial infiltration in the peri‐injury cortex increased significantly at day 3 post‐TBI compared to sham controls, while CPCGI treatment significantly reduced microglial infiltration compared to untreated TBI mice (TBI + vehicle vs. sham: 103.54 ± 12.22 vs. 56.56 ± 12.24; TBI + CPCGI vs. TBI + vehicle: 76.55 ± 11.92 vs. 103.54 ± 12.22; *p* < 0.05, Figure S1A,B). Further analysis showed that microglial endpoints per cell and process length per cell, indicators of microglial activation, were significantly reduced in the peri‐injury cortex of TBI mice compared to sham controls (endpoints, 31.55 ± 4.50 vs. 71.57 ± 6.45; process length, 325.34 ± 63.97 vs. 605.78 ± 82.88; *p* < 0.05, Figure 3A–C). CPCGI treatment led to increased endpoints and process length compared to the untreated TBI + vehicle group (endpoints, 41.18 ± 6.33 vs. 31.55 ± 4.50; process length, 411.11 ± 42.48 vs. 325.34 ± 63.97. *p* < 0.05, Figure 3A–C).

To further explore the effect of CPCGI on the polarization of microglia, pro‐inflammatory‐type markers (CD16, IL‐1β, IL‐18, IL‐6, and TNF‐α) and anti‐inflammatory‐type markers (CD206, TGF‐β, IL‐10, Arg‐1 and YM‐1) were analyzed by PCR at day 3 after injury. *CD16* expression was significantly upregulated in TBI mice compared to sham controls (3.30 ± 0.48 vs. 1.00 ± 0.12, *p* < 0.05). However, CPCGI treatment significantly downregulated the expressions of *CD16* (1.20 ± 0.19 vs. 3.30 ± 0.48, *p* < 0.05) and upregulated the expressions of *CD206* (3.04 ± 0.39 vs. 1.08 ± 0.15, *p* < 0.05) in injured mice compared with the untreated TBI + vehicle group (Figure 3D,E). As shown, the mRNA expressions of *IL‐1β* (7.68 ± 0.94 vs. 1.00 ± 0.27, *p* < 0.05), *IL‐18* (6.29 ± 0.90 vs. 1.00 ± 0.23, *p < 0.05*), *IL‐6* (13.93 ± 1.49 vs. 1.00 ± 0.31, *p* < 0.05), and *TNF‐α* (8.25 ± 1.20 vs. 1.00 ± 0.19, *p* < 0.05) were significantly upregulated in the TBI + vehicle group at day 3 compared with sham‐operated mice (Figure 3D). Compared with the TBI + vehicle group, CPCGI treatment significantly reduced the expressions of *IL‐1β* (3.16 ± 0.99 vs. 7.68 ± 0.94, *p* < 0.05), *IL‐18* (1.69 ± 0.67 vs. 6.29 ± 0.90, *p* < 0.05), *IL‐6* (5.60 ± 1.31 vs. 13.93 ± 1.49, *p* < 0.05), and *TNF‐α* (4.06 ± 0.91 vs. 8.25 ± 1.20, *p* < 0.05) at day 3 after experimental injury (Figure 3D). However, CPCGI treatment significantly elevated the levels of *TGF‐β* (2.19 ± 0.53 vs. 0.39 ± 0.18, *p* < 0.05), *IL‐10* (4.62 ± 0.82 vs. 1.46 ± 0.53, *p* < 0.05), *Arg‐1* (3.40 ± 0.82 vs. 0.69 ± 0.40, *p* < 0.05), and *YM‐1* (1.59 ± 0.62 vs. 0.60 ± 0.34, *p* < 0.05) compared to the TBI + vehicle group on day 3 following the experimental injury (Figure 3E).

Microglia regulate the inflammatory environment by secreting cytokines. The concentrations of IL‐1β (59.30 ± 5.68 vs. 0.76 ± 0.46, *p* < 0.05), IL‐18 (135.57 ± 35.94 vs. 1.47 ± 0.59, *p* < 0.05), IL‐6 (2198.16 ± 505.57 vs. 1.52 ± 1.71, *p* < 0.05), and TNF‐α (1598.07 ± 170.68 vs. 2.70 ± 1.93, *p* < 0.05) in the plasma samples were increased when exposed to traumatic brain injury, and CPCGI treatment reversed the upregulation of IL‐1β (16.45 ± 2.95 vs. 59.30 ± 5.68, *p* < 0.05), IL‐18 (60.96 ± 4.61 vs. 135.57 ± 35.94, *p* < 0.05), IL‐6 (187.71 ± 51.69 vs. 2198.16 ± 505.57, *p* < 0.05), and TNF‐α (195.47 ± 92.75 vs. 1598.07 ± 170.68, *p* < 0.05) induced by cerebral trauma compared with the TBI + vehicle group (Figure 3F). Collectively, these findings demonstrate that CPCGI effectively mitigates microglia‐mediated neuroinflammation after injury.

### 3.3 CPCGI Suppressed Pyroptosis in the Peri‐Injury Cortex After TBI

**FIGURE 4:**
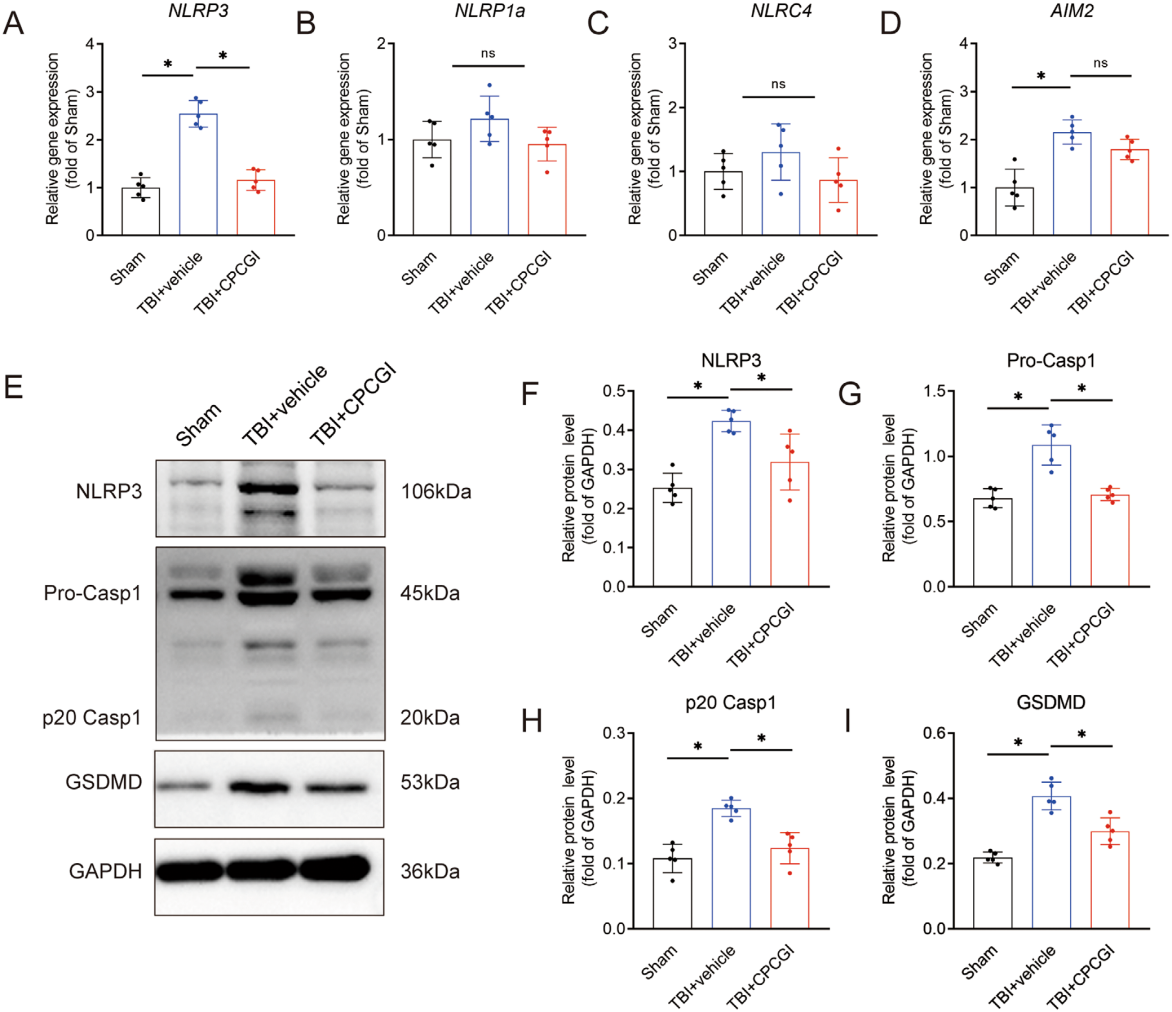
CPCGI suppressed pyroptosis in the peri‐infarct cortex after TBI. (A) Relative gene expression of NLRP3. *p* < 0.001 (TBI + vehicle vs. Sham), *p* = 0.000001 (TBI + CPCGI vs. TBI + vehicle); one‐way ANOVA followed by LSD multiple comparison tests. *n* = 5. (B) Relative gene expression of NLRP1a. *n* = 5. (C) Relative gene expression of NLRC4. *n* = 5. (D) Relative gene expression of AIM2. *p* = 0.000041 (TBI + vehicle vs. Sham); one‐way ANOVA followed by LSD multiple comparison tests. *n* = 5. (E) The protein levels of NLRP3, pro‐caspase‐1, p20 caspase‐1 and GSDMD in each group. (F) Relative protein levels of NLRP3. *p* = 0.000140 (TBI + vehicle vs. Sham), *p* = 0.005723 (TBI + CPCGI vs. TBI + vehicle); one‐way ANOVA followed by LSD multiple comparison tests. *n* = 5. (G) Relative protein levels of pro‐caspase‐1. *p* = 0.005529 (TBI + vehicle vs. Sham), *p* = 0.010077 (TBI + CPCGI vs. TBI + vehicle); one‐way ANOVA followed by Dunnett's multiple comparison tests. *n* = 5. (H) Relative protein levels of p20 caspase‐1. *p* = 0.000059 (TBI + vehicle vs. Sham), *p* = 0.000434 (TBI + CPCGI vs. TBI + vehicle); one‐way ANOVA followed by LSD multiple comparison tests. *n* = 5. (I) Relative protein levels of GSDMD. *p* = 0.000002 (TBI + vehicle vs. Sham), *p* = 0.000405 (TBI + CPCGI vs. TBI + vehicle); one‐way ANOVA followed by LSD multiple comparison tests. *n* = 5.

Inflammasomes, known to mediate the maturation of IL‐1β and IL‐18 and induce pyroptosis, have been implicated in TBI pathogenesis [8]. Cytosolic pattern recognition receptors (PRRs) are important components of the inflammasome, which induce oligomerization of sensors by recognizing inflammatory mediators and damage‐associated molecular patterns (DAMPs), mainly including *NLRP1a*, *NLRP3*, *NLRC4*, or *AIM2* [21]. We assessed mRNA expression levels of *NLRP3*, *NLRP1a*, *NLRC4*, and *AIM2* at day 3 post‐injury. Compared to the sham group, mRNA levels of *NLRP3* (2.55 ± 0.28 vs. 1.00 ± 0.21, *p* < 0.05) and *AIM2* (2.16 ± 0.25 vs. 1.00 ± 0.38, *p* < 0.05) significantly elevated following TBI, while *NLRP1a* and *NLRC4* levels remained unchanged (Figure 4A–D). Interestingly, CPCGI treatment significantly reduced *NLRP3* mRNA expression following injury (1.16 ± 0.21 vs. 2.55 ± 0.28, *p* < 0.05) but had no effect on *AIM2* levels (Figure 4A,D).

NLRP3 activation is central to the release of pro‐inflammatory factors in microglia and plays a crucial role in TBI‐related neuroinflammation. To determine the effects of CPCGI on NLRP3 inflammasome activation, western blot analysis was performed. TBI‐induced elevations in protein levels of NLRP3 (0.42 ± 0.03 vs. 0.25 ± 0.04, *p* < 0.05), pro‐caspase‐1 (1.09 ± 0.15 vs. 0.68 ± 0.07, *p* < 0.05), and p20 caspase‐1 (0.18 ± 0.01 vs. 0.11 ± 0.02, *p* < 0.05) were observed, confirming NLRP3 inflammasome activation (Figure 4E–H). As predicted, the CPCGI treatment reversed the upregulation of NLRP3 (0.32 ± 0.07 vs. 0.42 ± 0.03, *p* < 0.05), pro‐caspase‐1 (0.71 ± 0.05 vs. 1.09 ± 0.15, *p* < 0.05), and p20 caspase‐1 (0.12 ± 0.02 vs. 0.18 ± 0.01, *p* < 0.05) expressions induced by cerebral trauma compared with the TBI + vehicle group (Figure 4E–H). Activation of the NLRP3 inflammasome also leads to cleavage of full‐length gasdermin D (GSDMD), producing the GSDMD N‐terminus, a key pyroptosis effector [15]. Following TBI, GSDMD protein levels increased significantly (0.41 ± 0.04 vs. 0.22 ± 0.02, *p* < 0.05), an effect notably inhibited by CPCGI treatment (0.30 ± 0.04 vs. 0.41 ± 0.04, *p* < 0.05, Figure 4E,I). Collectively, these findings showed that CPCGI inhibited pyroptosis in TBI mice by preventing the activation of NLRP3 inflammasome.

### 3.4 CPCGI Improved Inflammatory Microenvironment Induced by Activated Microglia Cells In Vitro

**FIGURE 5:**
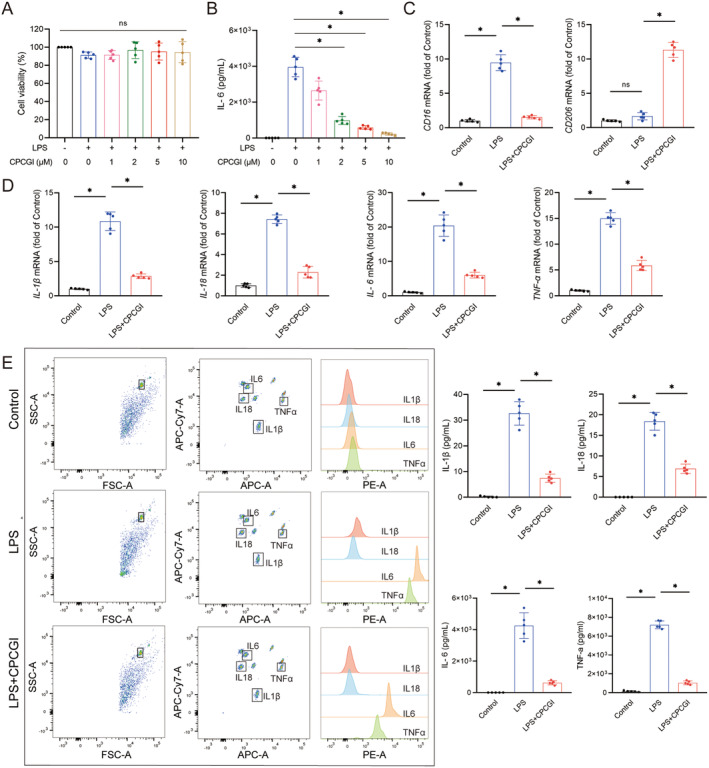
CPCGI improved inflammatory microenvironment induced by activated microglia cells in vitro. (A) CCK‐8 was used to assess BV2 cell viability. (B) The concentrations of IL‐6. *p* = 0.000655 (LPS vs. Control), *p* = 0.000615 (CPCGI 2 μM vs. LPS), *p* = 0.000896 (CPCGI 5 μM vs. LPS), *p* = 0.000693 (CPCGI 10 μM vs. LPS) by one‐way ANOVA followed by Dunnett's multiple comparison tests. *n* = 5. (C) The mRNA expression of CD16 and CD206. CD16: *P* = 0.000155 (LPS vs. Control), *p* = 0.000171 (LPS + CPCGI vs. LPS); CD206: *P* = 0.000009 (LPS + CPCGI vs. LPS); one‐way ANOVA followed by Dunnett's multiple comparison tests. *n* = 5. (D) The mRNA expression of IL‐1β, IL‐18, IL‐6, and TNF‐α. IL‐1β, *p* = 0.000201 (LPS vs. Control), *p* = 0.000263 (LPS + CPCGI vs. LPS); IL‐18, *p* = 0.000001 (LPS vs. Control), *p* = 0.000001 (LPS + CPCGI vs. LPS); IL‐6, *p* = 0.000377 (LPS vs. Control), *p* = 0.000738 (LPS + CPCGI vs. LPS); TNF‐α, *p* < 0.001 (LPS vs. Control), *p* < 0.001 (LPS + CPCGI vs. LPS); one‐way ANOVA followed by Dunnett's multiple comparison tests (IL‐1β, IL‐18 and IL‐6) or LSD multiple comparison tests (TNF‐α). *n* = 5. (E) The concentrations of IL‐1β, IL‐18, IL‐6, and TNF‐α. IL‐1β, *p* = 0.000229 (LPS vs. Control), *p* = 0.000229 (LPS + CPCGI vs. LPS); IL‐18, *p* = 0.000116 (LPS vs. Control), *p* = 0.000119 (LPS + CPCGI vs. LPS); IL‐6, *p* = 0.000796 (LPS vs. Control), *p* = 0.001218 (LPS + CPCGI vs. LPS); TNF‐α, *p* = 0.000004 (LPS vs. Control), *p* < 0.001 (LPS + CPCGI vs. LPS); one‐way ANOVA followed by Dunnett's multiple comparison tests. *n* = 5.

Given the observed anti‐inflammatory effects of CPCGI in vivo, we investigated whether CPCGI could similarly modulate microglia‐mediated inflammation in an in vitro model of TBI. We used an LPS‐induced microglial activation model to simulate the trauma‐induced neuroinflammatory environment, as previously described [19]. BV2 microglial cells were treated with CPCGI at concentrations ranging from 1 to 10 μM, and cell viability was assessed. Results indicated that CPCGI did not induce cytotoxicity in LPS‐treated BV2 cells (Figure 5A). To identify the optimal concentration for CPCGI, we assessed IL‐6 levels, which were significantly elevated by LPS stimulation. CPCGI treatment reduced IL‐6 in a dose‐dependent manner, with a 5 μM concentration selected for subsequent experiments (CPCGI 2 μM vs. LPS: 978.99 ± 220.68 vs. 3956.21 ± 539.43; CPCGI 5 μM vs. LPS: 584.15 ± 101.71 vs. 3956.21 ± 539.43; CPCGI 10 μM vs. LPS: 226.08 ± 72.41 vs. 3956.21 ± 539.43; *p* < 0.05, Figure 5B).

Further, LPS significantly upregulated the mRNA expressions of *CD16* in BV2 cells (9.46 ± 1.15 vs. 1.00 ± 0.18; *p* < 0.05, Figure 5C). CPCGI treatment significantly attenuated this alteration of *CD16* (1.52 ± 0.24 vs. 9.46 ± 1.15, *p* < 0.05) and increased the mRNA expression of *CD206* (11.32 ± 1.11 vs. 1.66 ± 0.53, *p* < 0.05) in BV2 cells stimulated by LPS (Figure 5C). Similarly, cytokine profiling showed that LPS stimulation significantly increased mRNA levels of *IL‐1β*, *IL‐18*, *IL‐6*, and *TNF‐α* in BV2 cells compared to controls (*IL‐1β*: 10.86 ± 1.35 vs. 1.00 ± 0.10; *IL‐18*: 7.43 ± 0.41 vs. 1.00 ± 0.18; *IL‐6*: 20.38 ± 3.10 vs. 1.00 ± 0.19; *TNF‐α*: 14.98 ± 1.12 vs. 1.00 ± 0.15; *p* < 0.05, Figure 5D). CPCGI treatment significantly reduced the mRNA expressions of *IL‐1β* (2.85 ± 0.35 vs. 10.86 ± 1.35, *p* < 0.05), *IL‐18* (2.28 ± 0.56 vs. 7.43 ± 0.41, *p* < 0.05), *IL‐6* (6.01 ± 0.83 vs. 20.38 ± 3.10, *p* < 0.05), and *TNF‐α* (5.85 ± 0.99 vs. 14.98 ± 1.12, *p* < 0.05) in BV2 cells stimulated by LPS (Figure 5D). In the supernatant, concentrations of IL‐1β, IL‐18, IL‐6, and TNF‐α were also markedly elevated by LPS stimulation compared to control conditions (IL‐1β: 32.63 ± 4.57 vs. 0.17 ± 0.26; IL‐18: 18.39 ± 2.16 vs. 0; IL‐6: 4255.95 ± 815.39 vs. 0.39 ± 0.40; TNF‐α: 7205.73 ± 412.33 vs. 135.38 ± 77.24; *p* < 0.05, Figure 5E). CPCGI treatment reversed these elevations (IL‐1β: 7.39 ± 1.60 vs. 32.63 ± 4.57; IL‐18: 6.89 ± 1.12 vs. 18.39 ± 2.16; IL‐6: 623.44 ± 129.85 vs. 4255.95 ± 815.39; TNF‐α: 1049.35 ± 202.84 vs. 7205.73 ± 412.33; *p* < 0.05, Figure 5E). These results indicate that CPCGI has a potent anti‐inflammatory effect on LPS‐induced microglial activation, suggesting its therapeutic potential in neuroinflammatory conditions.

### 3.5 CPCGI Inhibited NLRP3 Inflammasome‐Mediated Pyroptosis In Vitro

**FIGURE 6:**
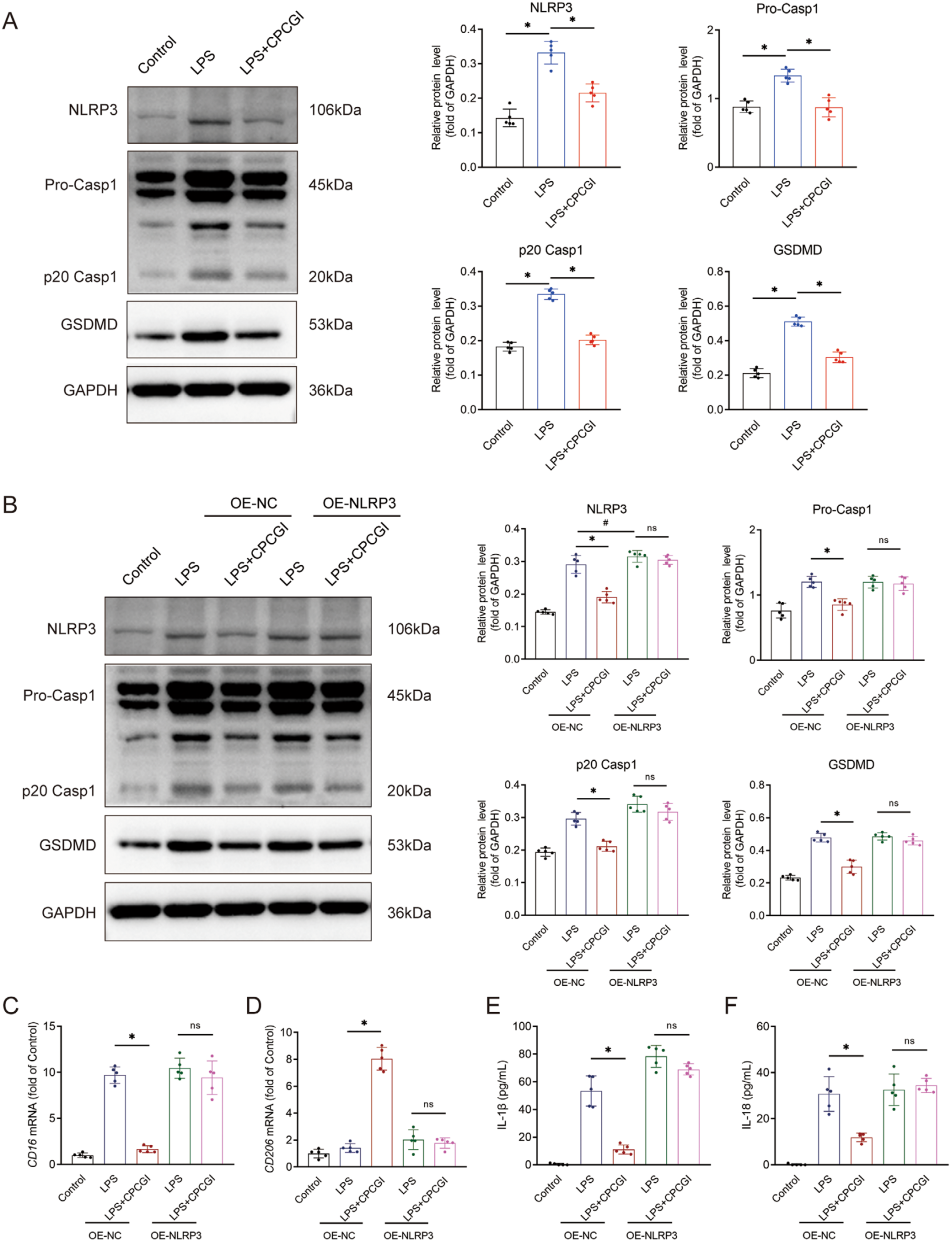
CPCGI inhibited NLRP3 inflammasome‐mediated pyroptosis in microglia cells stimulated by LPS. (A) The protein levels of NLRP3, pro‐caspase‐1, p20 caspase‐1 and GSDMD in each group. NLRP3, *p* < 0.001 (LPS vs. Control), *p* = 0.00003 (LPS + CPCGI vs. LPS); pro‐caspase‐1, *p* = 0.000026 (LPS vs. Control), *p* = 0.000022 (LPS + CPCGI vs. LPS); p20 caspase‐1, *p* < 0.001 (LPS vs. Control), *p* < 0.001 (LPS + CPCGI vs. LPS); GSDMD, *p* < 0.001 (LPS vs. Control), *p* < 0.001 (LPS + CPCGI vs. LPS); one‐way ANOVA followed by LSD multiple comparison tests. *n* = 5. (B) The protein levels of NLRP3, pro‐caspase‐1, p20 caspase‐1 and GSDMD in each group. NLRP3, *p < 0.001 (OE‐NC LPS + CPCGI vs. OE‐NC LPS), #*p* = 0.043562 (OE‐NLRP3 LPS vs. OE‐NC LPS); pro‐caspase‐1, *p* = 0.000015 (OE‐NC LPS + CPCGI vs. OE‐NC LPS); p20 caspase‐1, *p* = 0.000002 (OE‐NC LPS + CPCGI vs. OE‐NC LPS); GSDMD, *p* < 0.001 (OE‐NC LPS + CPCGI vs. OE‐NC LPS); one‐way ANOVA followed by LSD multiple comparison tests. *n* = 5. (C) The mRNA expression of CD16. *p* = 0.000034 (OE‐NC LPS + CPCGI vs. OE‐NC LPS) by one‐way ANOVA followed by Dunnett's multiple comparison tests. *n* = 5. (D) The mRNA expression of CD206. *p* < 0.001 (OE‐NC LPS + CPCGI vs. OE‐NC LPS) by one‐way ANOVA followed by LSD multiple comparison tests. *n* = 5. (E) The concentrations of IL‐1β. *p* = 0.003757 (OE‐NC LPS + CPCGI vs. OE‐NC LPS) by one‐way ANOVA followed by Dunnett's multiple comparison tests. *n* = 5. (F) The concentrations of IL‐18. *p* = 0.025382 (OE‐NC LPS + CPCGI vs. OE‐NC LPS) by one‐way ANOVA followed by Dunnett's multiple comparison tests. *n* = 5.

To elucidate the mechanism by which CPCGI mitigates microglial pyroptosis following brain trauma, we assessed the expression of key inflammasome‐related proteins, including NLRP3, pro‐caspase‐1, p20 caspase‐1, and GSDMD, using western blot analysis. As anticipated, LPS stimulation significantly increased the expression levels of NLRP3 (0.33 ± 0.03 vs. 0.14 ± 0.03, *p* < 0.05), pro‐caspase‐1 (1.34 ± 0.09 vs. 0.88 ± 0.08, *p < 0.05*), p20 caspase‐1 (0.33 ± 0.02 vs. 0.18 ± 0.01, *p* < 0.05), and GSDMD (0.51 ± 0.03 vs. 0.21 ± 0.03, *p* < 0.05) in LPS‐stimulated microglia compared to controls (Figure 6A). Moreover, CPCGI treatment significantly reduced the expressions of NLRP3 (0.22 ± 0.03 vs. 0.33 ± 0.03, *p* < 0.05), pro‐caspase‐1 (0.87 ± 0.14 vs. 1.34 ± 0.09), p20 caspase‐1 (0.20 ± 0.01 vs. 0.33 ± 0.02, *p* < 0.05), and GSDMD (0.30 ± 0.03 vs. 0.51 ± 0.03, *p* < 0.05) in microglia cells stimulated by LPS (Figure 6A).

Next, we overexpressed NLRP3 in BV2 cells via lentiviral infection. In vitro, NLRP3 overexpression was achieved by transducing lentivirus containing the NLRP3 gene (OE‐NLRP3), and lentivirus containing GFP (OE‐NC) was used as a control (Figure S2A,B). In the OE‐NC group, CPCGI treatment reduced LPS‐induced upregulation of NLRP3 (OE‐NC LPS + CPCGI vs. OE‐NC LPS: 0.19 ± 0.02 vs. 0.29 ± 0.03, *p* < 0.05), pro‐caspase‐1 (0.85 ± 0.09 vs. 1.20 ± 0.08), p20 caspase‐1 (0.21 ± 0.02 vs. 0.30 ± 0.02, *p* < 0.05), and GSDMD (0.30 ± 0.04 vs. 0.48 ± 0.03, *p* < 0.05) (Figure 6B). Notably, in the NLRP3‐overexpressing group (OE‐NLRP3), CPCGI's inhibitory effect on these protein levels was completely nullified (OE‐NLRP3 LPS + CPCGI vs. OE‐NLRP3 LPS, *p* > 0.05, Figure 6B).

Additionally, CPCGI treatment significantly attenuated LPS‐induced upregulation of *CD16* (OE‐NC LPS + CPCGI vs. OE‐NC LPS: 1.66 ± 0.38 vs. 9.70 ± 0.90, *p* < 0.05, Figure 6C) and increased the mRNA expression of *CD206* (OE‐NC LPS + CPCGI vs. OE‐NC LPS: 8.04 ± 0.84 vs. 1.41 ± 0.32, *p* < 0.05, Figure 6D) in the OE‐NC group. However, NLRP3 overexpression abolished these regulatory effects of CPCGI on *CD16* and *CD206* expression (OE‐NLRP3 LPS + CPCGI vs. OE‐NLRP3 LPS, *p* > 0.05, Figure 6C,D).

Furthermore, we assessed IL‐1β and IL‐18, key downstream cytokines of NLRP3 inflammasome activation. In the OE‐NC group, CPCGI significantly reduced LPS‐induced IL‐1β (OE‐NC LPS + CPCGI vs. OE‐NC LPS: 11.09 ± 3.31 vs. 53.37 ± 10.95, *p* < 0.05, Figure 6E) and IL‐18 (OE‐NC LPS + CPCGI vs. OE‐NC LPS: 11.89 ± 1.88 vs. 30.75 ± 7.54, *p* < 0.05, Figure 6F) levels in BV2 cells. Moreover, in the OE‐NLRP3 group, the suppressive effects of CPCGI on these cytokines were negated (OE‐NLRP3 LPS + CPCGI vs. OE‐NLRP3 LPS, *p* > 0.05, Figure 6E,F). Thus, these results suggested that CPCGI alleviated microglial pyroptosis by impeding NLRP3 inflammasome activation in LPS‐stimulated microglial cells.

### 3.6 CPCGI Mitigated Neural Damage by Inhibiting Microglial Pyroptosis Following TBI

**FIGURE 7:**
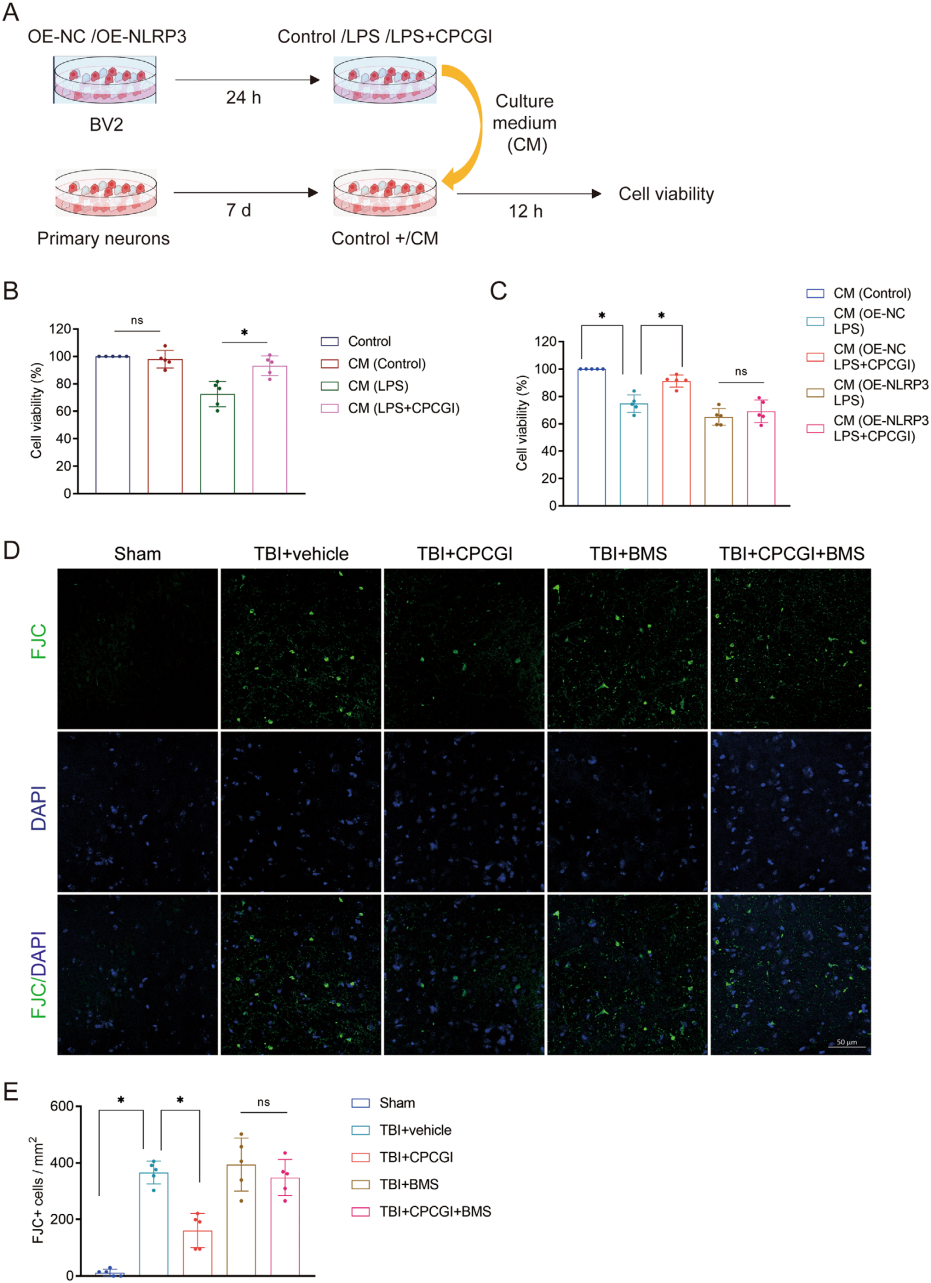
CPCGI mitigated neural damage through the inhibition of microglial pyroptosis following TBI. (A) Schematic diagram of the experimental design in vitro. (B) CCK‐8 was used to assess primary neurons cell viability. *p* = 0.007260 (CM (LPS) vs. CM (Control)), *p* = 0.024546 (CM (LPS + CPCGI) vs. CM (LPS)); one‐way ANOVA followed by Dunnett's multiple comparison tests. *n* = 5. (C) Cell viability of primary neurons. *p* = 0.005729 (CM (OE‐NC LPS) vs. CM (Control)), *p* = 0.016247 (CM (OE‐NC LPS + CPCGI) vs. CM (OE‐NC LPS)); one‐way ANOVA followed by Dunnett's multiple comparison tests. *n* = 5. (D) Representative pictures showing the results of FJC staining on 3 days post‐TBI. (E) Quantitative analysis of FJC positive cells per vision field. *p* = 0.000086 (TBI + vehicle vs. Sham), *p* = 0.003241 (TBI + CPCGI vs. TBI); one‐way ANOVA followed by Dunnett's multiple comparison tests. *n* = 5.

Considering the beneficial effects of CPCGI on microglial pyroptosis, we hypothesized that CPCGI might alleviate neuronal injury by modulating this process. To directly verify this hypothesis, we collected microglial supernatants from each group to make conditioned medium (CM) and further intervened in primary neurons (Figure 7A). Primary neurons were incubated in CM for 12 h, and cell viability was assessed using CCK‐8. Results indicated that CM co‐treated with LPS and CPCGI significantly enhanced neuronal viability compared to CM treated with LPS alone, suggesting a neuroprotective role of CPCGI mediated through regulation of microglial pyroptosis (CM(LPS + CPCGI) vs. CM(LPS): 93.21 ± 7.19 vs. 72.60 ± 9.20, *p* < 0.05, Figure 7B). Similar neuroprotective effects were observed in the CM (OE‐NC LPS + CPCGI) group (CM (OE‐NC LPS + CPCGI) vs. CM (OE‐NC LPS): 91.25 ± 4.42 vs. 74.71 ± 6.44, *p* < 0.05, Figure 7C). However, these protective effects were abrogated by NLRP3 overexpression (CM (OE‐NLRP3 LPS + CPCGI) vs. CM (OE‐NLRP3 LPS), *p* > 0.05, Figure 7C).

In parallel, neuronal degeneration in the peri‐injury cortex was evaluated 3 days post‐TBI using FJC staining. FJC‐positive neurons, indicative of degeneration, were significantly increased in the TBI + vehicle group compared to sham controls (TBI + vehicle vs. Sham: 365.96 ± 40.28 vs. 11.81 ± 12.35, *p* < 0.05, Figure 7D,E). CPCGI administration markedly reduced the number of FJC‐positive neurons compared to the TBI + vehicle group (TBI + CPCGI vs. TBI: 160.85 ± 60.26 vs. 365.96 ± 40.28, *p* < 0.05, Figure 7D,E). However, this neuroprotective effect was counteracted by BMS‐986299, a NLRP3 inflammasome agonist (TBI + CPCGI+BMS vs. TBI + BMS, *p* > 0.05, Figure 7D,E). Thus, the above results suggest that CPCGI mitigated neural damage through the inhibition of microglial pyroptosis following TBI.

## 4 Discussion

Traumatic brain injury disrupts both structural and functional integrity in brain tissue due to external forces, leading to significant global mortality and disability, with limited treatments for enhancing recovery. In this study, the role of microglial pyroptosis—a highly inflammatory form of programmed cell death—was explored as a pivotal mechanism contributing to secondary injury post‐TBI. Our findings indicate that CPCGI substantially enhances neurological recovery and reduces cerebral lesion volume in TBI models, underscoring its potential therapeutic value. Specifically, CPCGI was observed to inhibit microglial pyroptosis in the peri‐injury cortex post‐TBI, leading to a reduction in neuroinflammation and an improved inflammatory microenvironment. Mechanically, CPCGI appeared to suppress microglial pyroptosis by blocking NLRP3 inflammasome activation. When NLRP3 was overexpressed in microglia, the inhibitory effects of CPCGI on pyroptosis were reversed, highlighting CPCGI's specific modulation of NLRP3 inflammasome‐mediated microglial pyroptosis. Additionally, CPCGI was observed to alleviate neuronal injury by attenuating microglial pyroptosis both in vitro and in vivo, an effect nullified by either NLRP3 overexpression or activation with NLRP3 agonists. Collectively, these results suggest that CPCGI confers neuroprotection through the inhibition of NLRP3 inflammasome‐mediated microglial pyroptosis, positioning it as a promising therapeutic approach for TBI.

CPCGI, composed of peptides, gangliosides, and hypoxanthine, promoted neurological repair following various types of brain damage [29, 32, 34]. CPCGI had shown neuroprotective effects by inhibiting mitochondrial apoptotic signaling and the PARP/NF‐κB inflammatory pathway in TBI [30]. Furthermore, CPCGI protected against gray and white matter damage in the TBI model, likely through Nrf2 signaling activation and reduction of oxidative stress‐related calpain activation [32]. In cerebral ischemia, CPCGI improved blood circulation and neurological function, while in Alzheimer's disease (AD) mice, it had been reported to reduce amyloid‐beta accumulation, inhibit inflammation, and prevent neuronal apoptosis [28, 34]. Additionally, CPCGI exerts neuroprotective effects by regulating the MAPK/NF‐κB signaling pathway in models of sevoflurane‐induced neuronal damage [31]. Traumatic brain injury led to neuronal loss in the peri‐injury cortex of mice, a phenomenon also observed in human patients [45, 46]. Our findings demonstrated that CPCGI significantly improved behavioral scores following TBI. Additionally, Nissl staining revealed that CPCGI reduced tissue loss and neuronal damage in the brain. These results suggest that CPCGI may confer neuroprotective effects, enhancing recovery and mitigating the extent of neuronal injury after TBI.

While previous research extensively documented the neuroprotective effects of CPCGI on various brain damages, its impact on microglia remained unexplored until now. Our study demonstrated that CPCGI significantly reduced microglia‐mediated neuroinflammation following brain injury and improved the inflammatory microenvironment. The neuroinflammatory response was identified as a crucial factor in secondary injury after TBI, which is a complex pathological process that exacerbated the initial damage, leading to neuronal cell death and disruption of the blood–brain barrier [9, 19, 42, 47]. In the healthy brain, ramified microglia actively monitored the microenvironment, serving as essential mediators of the innate immune response in the central nervous system [48]. After TBI, resident microglia promptly responded to injury by detecting DAMPs and releasing cytokines and chemokines, thereby altering the inflammatory microenvironment [47, 49, 50]. Our findings revealed not only an increase in microglial numbers post‐TBI but also significant changes in their morphology and activation state. Microglia exhibited a debranching response, characterized by a reduction in the number of processes and a transition to an amoeboid shape, indicative of activation [51, 52, 53, 54]. CPCGI inhibited both the increase in microglial numbers and the associated morphological changes, suggesting a suppression of microglial activation. While activated microglia may confer benefits during the acute phase of TBI, prolonged activation could lead to detrimental effects [19, 55, 56]. To navigate the ongoing debate regarding M1 and M2 classifications, we referred to microglial phenotypes as pro‐inflammatory or anti‐inflammatory [44, 57]. In this study, CPCGI demonstrated significant inhibitory effects on neuroinflammation both in vivo and in vitro. It reduced the release of pro‐inflammatory cytokines, including CD16, IL‐1β, IL‐18, IL‐6, and TNF‐α, which are recognized as markers of the “pro‐inflammatory” microglial phenotype. Additionally, CPCGI partially enhanced the expression of certain markers associated with the “anti‐inflammatory” microglial phenotype, such as CD206 [17, 19, 44]. These findings suggest that CPCGI serves as a promising therapeutic target for the treatment of traumatic brain injury by modulating microglia‐mediated neuroinflammation.

Pyroptosis, an inflammatory form of cell death, was activated by DAMPs and pathogen‐associated molecular patterns (PAMPs) recognized by Toll‐like receptors (TLRs) and NOD‐like receptors (NLRs) on the membranes of neurons and glial cells. This process triggered the activation of inflammasomes, leading to the cleavage of GSDMD and the formation of pores in the cell membrane, which facilitated the release of pro‐inflammatory cytokines into the extracellular environment, exacerbating neuroinflammation and cerebral edema in affected brain tissues [16, 58, 59]. Recent studies identified cellular pyroptosis as a significant target in traumatic brain injury, particularly concerning secondary injury mechanisms following the initial trauma [2, 7, 9, 17]. Our findings supported this notion, revealing increased expression of GSDMD, pro‐caspase‐1, and p20 caspase‐1, along with elevated levels of IL‐1β and IL‐18 in the brain tissue of TBI mice, indicating the occurrence of pyroptosis post‐injury. Importantly, treatment with CPCGI effectively reversed these changes, confirming its inhibitory effects on pyroptosis. Inflammasomes are known to mediate the maturation of IL‐1β and IL‐18, leading to the induction of pyroptosis [16, 60]. Among these, the NLRP3 inflammasome is highly expressed in microglia and has been implicated in the pathogenesis of traumatic brain injury [8, 19, 22, 61]. However, the potential involvement of other inflammasomes, such as NLRP1, NLRC4, or AIM2, cannot be entirely dismissed, necessitating further investigation specifically targeting these inflammasomes [21]. Our results demonstrated that both NLRP3 and AIM2 expression levels were significantly upregulated following TBI, thereby extending the findings of previous studies. Notably, after CPCGI treatment, only NLRP3 activation was inhibited, suggesting that CPCGI specifically targeted NLRP3. The NLRP3 inflammasome, a multiprotein complex comprising NLRP3, ASC, and pro‐caspase‐1, was activated by microbial or danger‐associated signals, leading to caspase‐1 activation [62, 63, 64]. This process cleaved pro‐IL‐1β and pro‐IL‐18 for maturation and release, while also cleaving GSDMD to form membrane pores, facilitating the release of IL‐1β and IL‐18 and triggering pyroptosis [23, 59, 65, 66, 67, 68]. Our data indicated that following TBI, there was an upregulation of NLRP3 inflammasome‐related proteins, including NLRP3 and pro‐caspase‐1, which corroborated previous findings [2, 7, 8, 17]. Furthermore, both in vivo and in vitro experiments demonstrated that CPCGI effectively inhibited the activation of the NLRP3 inflammasome, associated pyroptosis, and the subsequent release of IL‐1β and IL‐18 following TBI. Interestingly, in microglial cells with NLRP3 gene overexpression, the inhibitory effect of CPCGI on NLRP3 inflammasome activation was abolished, along with its suppression of pyroptosis and downstream IL‐1β and IL‐18 release. This observation suggested that CPCGI exerts its protective effects by effectively inhibiting NLRP3 inflammasome‐mediated pyroptosis.

Microglia were essential for maintaining brain homeostasis under both physiological and pathological conditions, and their dysregulation is closely linked to the mechanisms of traumatic brain injury [55, 69, 70, 71]. In our study, conditioned medium from LPS‐activated microglia significantly reduced neuronal viability, supporting the hypothesis that inflammation induced by activated microglia contributes to neuronal damage following TBI [8, 19]. Importantly, treatment with CPCGI alleviated neuronal injury caused by this activated microglial supernatant, indicating that CPCGI effectively inhibited the neurotoxic effects of microglial activation. Moreover, conditioned medium derived from microglia overexpressing the NLRP3 gene reversed the protective effects of CPCGI, suggesting that CPCGI exerts its neuroprotective effects in vitro through the modulation of NLRP3 inflammasome‐mediated pyroptosis in microglia. This neuroprotective role of CPCGI was further corroborated in a TBI model with pharmacological activation of NLRP3 in vivo. Based on these results, we hypothesized that CPCGI regulates NLRP3 inflammasome‐mediated pyroptosis in microglia, thereby establishing a more favorable local microenvironment that mitigates neuronal injury following brain trauma. While we acknowledged the possibility of other mechanisms contributing to the modulation of microglial pyroptosis by CPCGI post‐TBI, our study expanded the application of CPCGI to microglial cells and elucidated a novel mechanism by which CPCGI exerts neuroprotective effects.

This study presented several limitations that necessitate further investigation. Although our controlled cortical impact model successfully replicated critical features of human traumatic brain injury, including pathological progression and neurological deficits, it did not assess various risk factors associated with TBI, such as hypertension, diabetes, age, and sex [6]. Furthermore, our research did not clarify the roles of other immune cells involved in neuroinflammation, particularly astrocytes. The potential beneficial effects of CPCGI on astrocyte‐mediated neuroinflammation warrant additional research. Addressing these limitations in future studies could provide a more comprehensive understanding of the intricate interactions among different cell types in the neuroinflammatory response following TBI and the therapeutic implications of CPCGI in modulating these responses.

## 5 Conclusions

In summary, we demonstrated that CPCGI effectively inhibited NLRP3 inflammasome activation‐mediated pyroptosis in microglia, both in vitro and in vivo, thereby improving the neuroinflammatory microenvironment and promoting neurological recovery following traumatic brain injury. Targeting NLRP3 inflammasome activation may represent a promising clinical strategy for TBI treatment, as it could alleviate neuroinflammation by regulating microglial pyroptosis, warranting further investigation. Furthermore, our research broadened the application of CPCGI to microglial cells and elucidated a novel mechanism through which CPCGI exerts its neuroprotective effects. These findings highlight the potential of CPCGI as a therapeutic agent in the management of TBI.

## Author Contributions

**Lu‐Lu Yu:** writing – original draft, resources, methodology, investigation, formal analysis, data curation, conceptualization. **Lei Sun:** resources, methodology, investigation, formal analysis, data curation, conceptualization. **Ting‐Ting Yu:** resources, methodology, investigation, formal analysis, data curation, conceptualization. **An‐Chen Guo:** writing – review and editing. **Jian‐Ping Wu:** writing – review and editing. **Jun‐Min Chen:** supervision, project administration, writing – review and editing. **Qun Wang:** supervision, project administration, funding acquisition, writing – review and editing.

## Conflicts of Interest

The authors declare no conflicts of interest.

## Supporting information


**Figure S1.** CPCGI ameliorated microglia‐mediated neuroinflammation after TBI. (A and B) Representative images of damaged cortex labeled with Iba1 (microglia marker) at 3 days after TBI and quantification of the Iba1+ cells in per mm^2^. *p* = 0.000007 (TBI + vehicle vs. sham); *p* = 0.001555 (TBI + CPCGI vs. TBI + vehicle); one‐way ANOVA followed by LSD multiple comparison tests. *n* = 6.

**Figure S2.** (A) Relative gene expression of NLRP3 following treatment with a lentiviral vector containing NLRP3. *p* = 0.001058 (OE‐NLRP3 vs. OE‐NC) by Student’s t‐test. *n* = 5. (B) The protein levels of NLRP3 in each group. *p* = 0.011734 (OE‐NLRP3 vs. OE‐NC) by Student’s t‐test. *n* = 5.

**Appendix S1.** Xxx.

## Data Availability

Data will be made available upon request.
