# Supplementary figures and images for: CPCGI Alleviates Neural Damage by Modulating Microglial Pyroptosis After Traumatic Brain Injury
Source: CNS Neurosci Ther. 2025 Mar 9;31(3):e70322. doi: 10.1111/cns.70322 (PMC11890976; doi:10.1111/cns.70322)

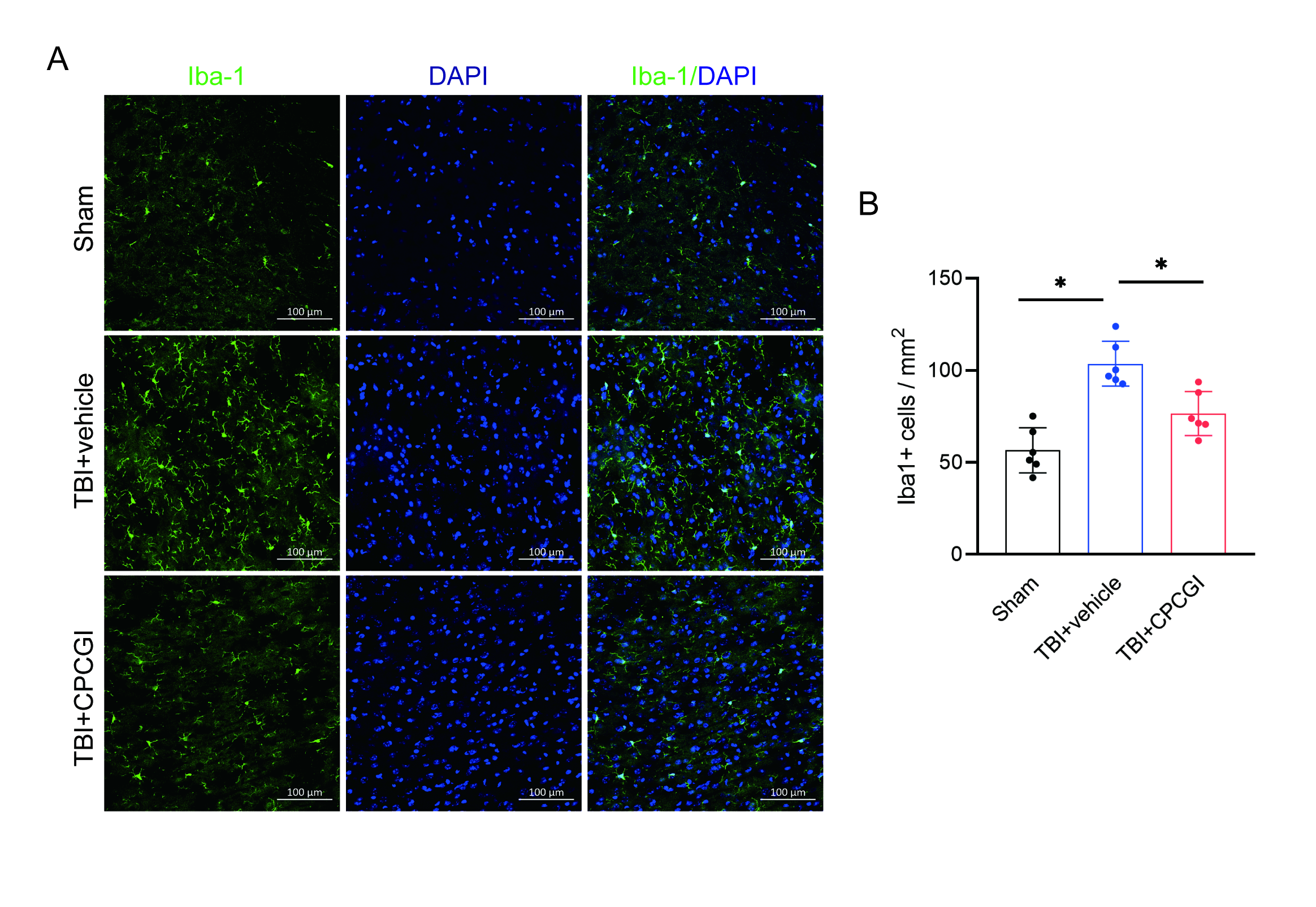

Supplement: Supplementary file 1 — Figure S1. CPCGI ameliorated microglia‐mediated neuroinflammation after TBI. (A and B) Representative images of damaged cortex labeled with Iba1 (microglia marker) at 3 days after TBI and quantification of the Iba1+ cells in per mm2. p = 0.000007 (TBI + vehicle vs. sham); p = 0.001555 (TBI + CPCGI vs. TBI + vehicle); one‐way ANOVA followed by LSD multiple comparison tests. n = 6. [file CNS-31-e70322-s003.tif]

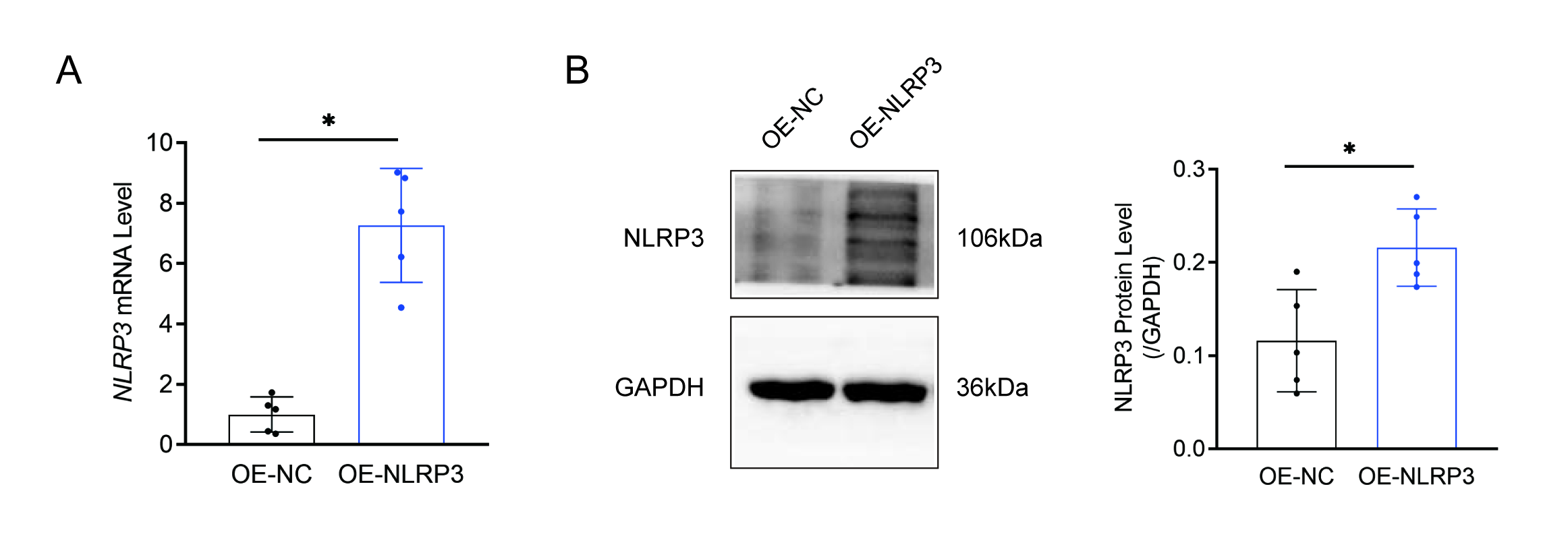

Supplement: Supplementary file 2 — Figure S2. (A) Relative gene expression of NLRP3 following treatment with a lentiviral vector containing NLRP3. p = 0.001058 (OE‐NLRP3 vs. OE‐NC) by Student’s t‐test. n = 5. (B) The protein levels of NLRP3 in each group. p = 0.011734 (OE‐NLRP3 vs. OE‐NC) by Student’s t‐test. n = 5. [file CNS-31-e70322-s002.tif]

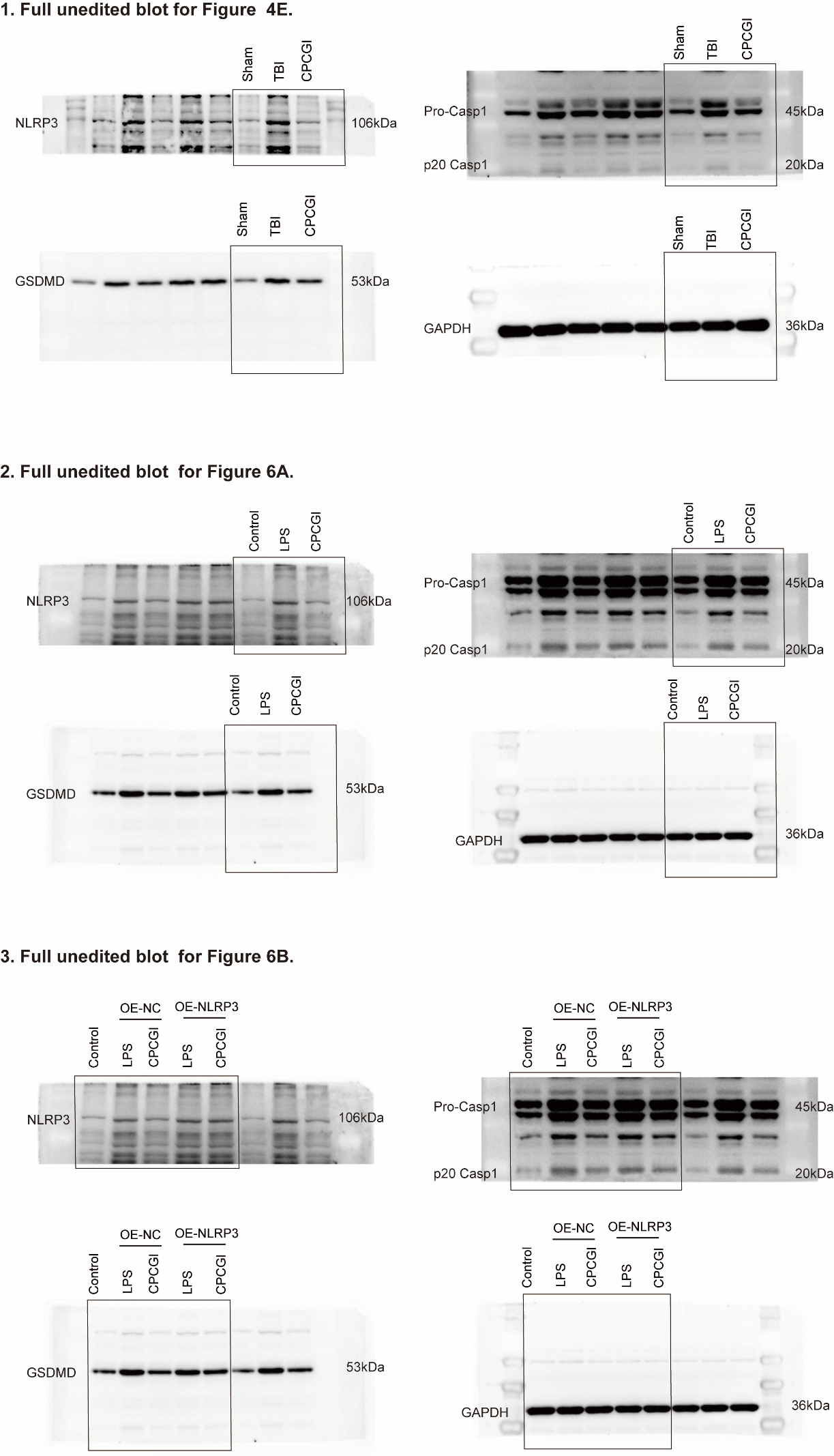

Supplement: Supplementary file 3 — Appendix S1. Xxx. [file CNS-31-e70322-s001.docx]
